# Clinical Outcomes of Immune Checkpoint Inhibitors in Unique Cohorts Underrepresented in Clinical Trials

**DOI:** 10.3390/cancers16122223

**Published:** 2024-06-14

**Authors:** Neil J. Shah, Alexandra Della Pia, Tianmin Wu, Aquino Williams, Melinda Weber, Brittany Sinclaire, Elli Gourna Paleoudis, Adil Alaoui, Shaked Lev-Ari, Shari Adams, Jordan Kaufman, Sahil B. Parikh, Emily Tonti, Eric Muller, Michael Serzan, Divya Cheruku, Albert Lee, Aishwarya Sridhar, Benjamin (Thor) Perrin Hee, Jaeil Ahn, Andrew Pecora, Andrew Ip, Michael B. Atkins

**Affiliations:** 1 Department of Medicine, Memorial Sloan Kettering Cancer Center, New York, NY 10065, USA; 2 Department of Medicine, Weill Cornell Medical Center, New York, NY 10065, USA; 3 John Theurer Cancer Center at Hackensack Meridian Health, Hackensack, NJ 07601, USA; 4 Department of Biostatistics, Georgetown University Medical Center, Washington, DC 20007, USA; 5 Hackensack Meridian Health Mountainside Medical Center, Montclair, NJ 07042, USA; 6 Department of Oncology, Georgetown Lombardi Comprehensive Cancer Center, Washington, DC 20007, USA; 7 Department of Medical Oncology, Dana-Farber Cancer Institute, Boston, MA 02215, USA; 8 Hackensack Meridian School of Medicine, Nutley, NJ 07110, USA

**Keywords:** immune checkpoint inhibitors (ICIs), immune-related adverse events (irAEs), pembrolizumab, nivolumab, non-small cell lung cancer (NSCLC)

## Abstract

**Simple Summary:**

Immune checkpoint inhibitors (ICIs) are a type of immunotherapy used to treat a variety of cancers by helping a patient’s own immune system to kill cancer cells. ICIs received their regulatory approval based on the results of large, randomized clinical trials. However, certain patient groups were excluded from these trials, so their outcomes are unknown. We performed a multicenter, retrospective study of real-world data in the United States in patients who had received at least one cycle of ICI treatment to evaluate the efficacy and safety of ICIs in patient groups underrepresented in clinical trials. Unique patient groups included age > 75 years, non-White race, positive smoking history, poor performance status, obesity, autoimmune diseases, chronic viral infections, multiple previous cancer therapies, or >three metastatic sites. Overall, ICIs were safe and efficacious in these patient groups. We noted that poor performance status and a history of multiple cancer therapies were associated with poor ICI efficacy, and Black patients, compared to White patients, experienced fewer immune-related adverse events.

**Abstract:**

Regulatory approval of immune checkpoint inhibitors (ICIs) was based on results of large, randomized clinical trials, resulting in limited outcomes data in patient cohorts typically underrepresented in such trials. The objective of this study was to evaluate the efficacy and safety of ICIs in these unique patient cohorts. This is a multicenter, retrospective analysis of real-world data at six academic and community clinics in the United States from 1 January 2011 to 1 April 2018. Patients were included if they had received at least one cycle of ICI treatment. Unique patient cohorts included age > 75 years, non-White race, positive smoking history, ECOG performance status (PS) ≥ 2, BMI ≥ 30 kg/m^2^, autoimmune diseases (AIDs), chronic viral infections (CVI), extensive prior lines of therapy (LOTs), or >three metastatic sites. Immune-related adverse events (irAEs), overall survival (OS), and time to treatment failure were evaluated in the entire cohort and in NSCLC patients treated with PD-(L)1 monotherapy. Outcomes and their association with unique patient cohorts were compared on univariate analysis and multivariate analysis to those without a particular characteristic in the entire NSCLC PD-(L)1 monotherapy cohorts. In total, 1453 patients were included: 56.5%—smokers, 30.4%—non-White, 22.8%—elderly, 20.8%—ECOG PS ≥ 2, 15.7%—history of AIDs, and 4.7%—history of CVI. The common ICIs were nivolumab (37.1%) and pembrolizumab (22.2%). Black patients, compared to White patients, experienced fewer irAEs (OR 0.54, *p* < 0.001). An ECOG PS of ≥2 (HR = 2.01, *p* < 0.001) and an increased number of previous LOTs were associated with poor OS (the median OS of 26.2 vs. 16.2 vs. 9.6 months for one vs. two vs. three prior LOTs, *p* < 0.001). The above results were confirmed in anti-PD-(L)1 monotherapy non-small cell lung cancer patients (n = 384). Overall, ICIs were safe and efficacious in these typically underrepresented patient cohorts. We noted ECOG PS ≥ 2 and an increased prior LOTs were associated with poor ICI efficacy, and Black patients, compared to White patients, experienced fewer irAEs.

## 1. Introduction

Immune checkpoint inhibitors (ICIs) have improved survival outcomes in a variety of solid tumors, including non-small cell lung cancer (NSCLC), melanoma, kidney, bladder, and gastrointestinal cancers [1,2,3,4,5,6,7,8,9,10]. The available ICIs received numerous FDA-approved indications based on robust, often randomized, multicenter clinical trial data. However, patients with poor Eastern Cooperative Oncology Group (ECOG) performance status (PS), autoimmune diseases (AID), chronic viral infections (CVI, including human immunodeficiency virus (HIV), Hepatitis B (HBV), Hepatitis C (HCV)), and of diverse race, were either excluded or underrepresented in such trials, limiting the generalizability of ICI therapy to a broader patient population. This underrepresentation has led to a need for real-world data for such patient cohorts to determine whether they may benefit from ICIs similarly to the more frequently enrolled populations and more than from alternative options.

Several retrospective studies and some prospective studies have tried to address the impact of ICIs in the unrepresented patient populations above. The prospective studies CheckMate 153 [11] and CheckMate 171 [12] and several retrospective studies have shown similar efficacy and safety for ICIs in elderly patients [13,14,15,16]. An ECOG PS ≥ 2 was associated with poor ICI outcomes in CheckMate 153, 171, and a meta-analysis performed by Tomasik et al. [11,12,17]. A few small retrospective studies suggested no difference in overall survival (OS) between Black and White patients [18,19]. Conflicting data exist regarding obesity and ICI outcomes. Animal studies [20] and other retrospective studies are suggestive of improved ICI efficacy and increased ICI toxicity among obese patients compared to those with a normal body mass index (BMI) [21,22,23,24]. However, in patients with melanoma, retrospective studies and a meta-analysis failed to demonstrate any association [25,26]. A few retrospective studies have shown similar ICI efficacy and toxicity in patients with underlying AIDs vs. no AIDs [27,28,29,30]. In addition, retrospective studies of patients with CVI, including HIV, HBV, and HCV, have shown similar ICI efficacy and safety compared to patients without CVI [31,32,33].

As the indications for ICIs continue to expand, there is an urgent need to understand the differences regarding the efficacy and safety of ICIs in clinically challenging patient populations historically excluded from clinical trials. We conducted a comprehensive analysis of real-world data (RWD) compiled from the patients treated at academic and community clinics in diverse geographic locations in the Eastern United States (US). The main objectives were to evaluate the efficacy and safety of ICIs among underrepresented patients in the entire cohort and in the NSCLC cohort and compare them to a more represented patient population receiving the same treatments and NSCLC patients treated with PD-(L)1 monotherapy, respectively.

## 2. Materials and Methods

### 2.1. Study Design

This was a multicenter, retrospective study of adult patients who received ICI treatment at 5 MedStar Health hospitals and Hackensack University Medical Center from January 2011 to April 2018. The 6 academic and community clinics included in this study represent populations in urban and suburban areas in the Eastern US. Institutional Review Board (IRB) approval was obtained under Georgetown University Medical Center IRB 2017-0559. This trial was conducted under the International Conference on Harmonization Good Clinical Practice guidelines and according to the Declaration of Helsinki. The requirement for patient informed consent (verbal or written) was waived by the IRB as this project represented a non-interventional study utilizing routinely collected data for secondary research purposes.

A comprehensive REDCap database was developed for data collection, and structured data were captured from the electronic health record (EHR) system using SQL queries. Manual data extraction was performed for non-structured data. AIDs was defined as a diagnosis of a variety of conditions, such as Hashimoto’s disease, hypothyroidism, etc. (Supplementary Text S1). Combined CVI included a history of HIV, HBV, and/or HCV.

### 2.2. Patients and Outcomes

All patients had received at least 1 dose of anti-PD-1, anti-PD-(L)1, anti-CTLA-4, or combination (e.g., anti-CTLA-4 and anti-PD-1; anti-PD-1 and chemotherapy) ICI therapy. ICI treatment was administered at standard doses, as per the recommendations of the United States (US) Federal Drug Administration (FDA), in the monotherapy or combination settings. Patients were assigned to the following cohorts: the overall population (entire cohort), all patients with NSCLC, and patients with NSCLC treated only with anti-PD-(L)1 monotherapy (Supplementary Figure S1). Within these cohorts, unique patient groups of interest included the following: age > 75 years, non-White race, positive smoking history, ECOG PS ≥ 2, BMI ≥ 30 kg/m^2^, AIDs, combined CVI, extensive prior lines of therapy (LOTs), or >3 metastatic sites. The non-White race patient group included all patients with Black, Asian, or other as their self-identified race and excluded Hispanic and non-Hispanic White patients, as ethnicity data were not available. Patients in the entire cohort were excluded if they received ICI treatment in the adjuvant or neoadjuvant settings. Patients in the NSCLC cohort were excluded if they received ICI treatment for small cell tumors or those with neuroendocrine features.

The Common Terminology of Adverse Events (CTCAE) v4.03 [34] was used to capture immune-related adverse events (irAEs) types and grades. Investigator-assessed real-world clinical outcomes were collected and included the following: OS, time to treatment failure (TTF), and overall response rate (ORR) (defined as complete response (CR) and partial response (PR)). These outcomes were assessed in unique cancer types, including the entire cohort, all patients with NSCLC, and patients with NSCLC treated only with anti-PD-(L)1 monotherapy (Supplementary Figure S1). OS was defined as the time from the start date of ICI to the date of death or last follow-up. TTF was defined as the time from the start date of ICI to the start date of the following LOT or death, whichever happened sooner. ORR was based on the RECIST v1.1 criteria or the treating investigator’s documentation.

### 2.3. Statistical Analysis

The baseline characteristics, clinical efficacy (e.g., time to event outcomes), and safety (e.g., irAEs) were descriptively summarized. For the incidence of irAEs, univariate analyses (UA) such as Pearson’s chi-squared test or Fisher’s exact test were initially conducted, as appropriate, to assess the association between baseline variables and the incidence of any grade irAEs and grade ≥ 3 irAEs. Age, race, smoking history, ECOG PS, BMI, AID, combined CVI, prior LOTs, and number of metastatic sites were used for UA. Multivariable logistic regression analyses were followed adjusting for age, sex, race, and the variables with *p*-value < 0.2 in the UA where odds ratios (OR) and 95% confidence intervals (CI) were summarized. The omnibus goodness-of-fit tests were used to evaluate the fit of multivariable logistic models. For the efficacy analyses, median OS and TTF were estimated by the Kaplan–Meier (KM) method, and the log-rank test was used to evaluate differences among unique patient cohorts. Multivariable adjusted Cox models were adjusted for all variables in the UA. The May and Hosmer goodness-of-fit tests were conducted for the multivariable-adjusted Cox models [35]. A two-sided *p*-value < 0.05 was considered statistically significant for the safety endpoint, and Bonferroni multiplicity adjusted *p* < 0.0167 (0.05/3) can also be considered for overall endpoints. All analyses were conducted using R software (v.4.3; R Core Team, Vienna, Austria) [36].

## 3. Results

### 3.1. Patient Characteristics

#### 3.1.1. Entire Cohort

We identified 1453 patients who received ICI therapy, including 34.4% lung cancer, 27.8% melanoma, and 7.2% gastrointestinal cancers (Table 1). The median age was 65.9 years (IQR 56.6, 74.4), 69.6% were White, and 57.8% were male. The unique patient groups of interest included 56.5% with a smoking history, 30.4% non-White, 22.8% elderly, 20.8% with ECOG PS ≥ 2, 15.7% with a history of AIDs, and 4.7% with a history of combined CVI. The common ICI treatments were nivolumab (37.1%), pembrolizumab (22.2%), nivolumab plus ipilimumab (13.2%), and ipilimumab (11.2%).

#### 3.1.2. Non-Small Cell Lung Cancer Cohort

Of the 499 patients with lung cancer, 443 patients were diagnosed with NSCLC (Supplementary Table S1), including 384 who received anti-PD-(L)1 monotherapy (Table 1). In the anti-PD-(L)1 monotherapy NSCLC patients, the median age was 70.1 years (IQR 61.8, 76.3), 59.1% were White, and 50.8% were male. Unique patient groups included 40.9% non-White, 31.1% elderly, 25.9% with ECOG PS ≥ 2, 83.1% with a smoking history, 15% obese, 14.1% with a history of AID, and 4.2% with a history of combined CVI. Common anti-PD-(L)1 treatments were nivolumab (63.8%) and pembrolizumab (26.8%).

### 3.2. Safety Analysis

#### 3.2.1. Entire Cohort

The incidence of any grade and grade ≥ 3 irAEs was 39.3% and 12.6%, respectively. In the univariate analysis, among unique patient cohorts, race (*p* < 0.001), obesity (*p* = 0.004), and ECOG PS ≥ 2 (*p* < 0.001) for any grade irAEs were significant (Supplementary Table S2). In the multivariate analysis, Black patients were less likely to experience any grade (OR 0.54, *p* < 0.001) or grade ≥ 3 irAEs (OR 0.49, *p* = 0.008) compared to White patients. Similar results were noted for Asian patients. Obese patients were more likely to experience any grade (OR 1.44, *p* = 0.006) and grade ≥ 3 irAEs (OR 1.61, *p* = 0.007) when compared to non-obese (BMI < 30 mg/kg^2^) patients. Patients with an ECOG PS ≥ 2 developed less any grade (OR 0.46, *p* < 0.001) or grade ≥ 3 irAEs (OR 0.45, *p* < 0.001) compared to patients with ECOG PS < 2 (Table 2).

#### 3.2.2. Anti-PD-(L)1 Monotherapy Non-Small Cell Lung Cancer Cohort

The incidence of any grade and grade ≥ 3 irAEs was 30.5% and 8.1%, respectively. In univariate analysis, irAEs among unique patient cohorts—elderly (*p* = 0.045), White (*p* = 0.026), female (*p* = 0.026), history of combined CVI (*p* = 0.048), history of AIDs (*p* = 0.026), and ECOG PS < 2 (*p* = 0.045, Supplementary Table S5)—were significant compared to patients without a particular characteristic. On multivariate analysis, Black patients vs. White (OR 0.53, *p* = 0.023), those without a history of combined CVI vs. with CVI (OR 0.22, *p* = 0.006), and with ECOG PS ≥ 2 vs. ECOG 0–1 (OR 0.55, *p* = 0.036) appeared less likely to experience any grade irAEs (Table 3). In contrast, patients with a history of AIDs were more likely to develop any grade irAEs (OR 1.93, *p* = 0.037) compared to those without AIDs. Similar findings for these underrepresented populations relative to the other patients were observed in the entire NSCLC Cohort (ICI monotherapy plus ICI + chemotherapy, n = 443, Supplementary Tables S3 and S4). On multivariate analysis, White patients with an ECOG PS ≥ 2 developed less any grade (OR 0.35, *p* = 0.008) irAEs compared to patients with ECOG PS < 2 (Supplementary Table S6). Differently, Black patients without a history of combined CVI were less likely to develop any grade (OR 0.18, *p* = 0.028) or grade ≥ 3 (OR 0.03, *p* = 0.010) irAEs compared to patients with a history of combined CVI, (Supplementary Table S7).

### 3.3. Efficacy Analysis

#### 3.3.1. Entire Cohort

**Figure 1:**
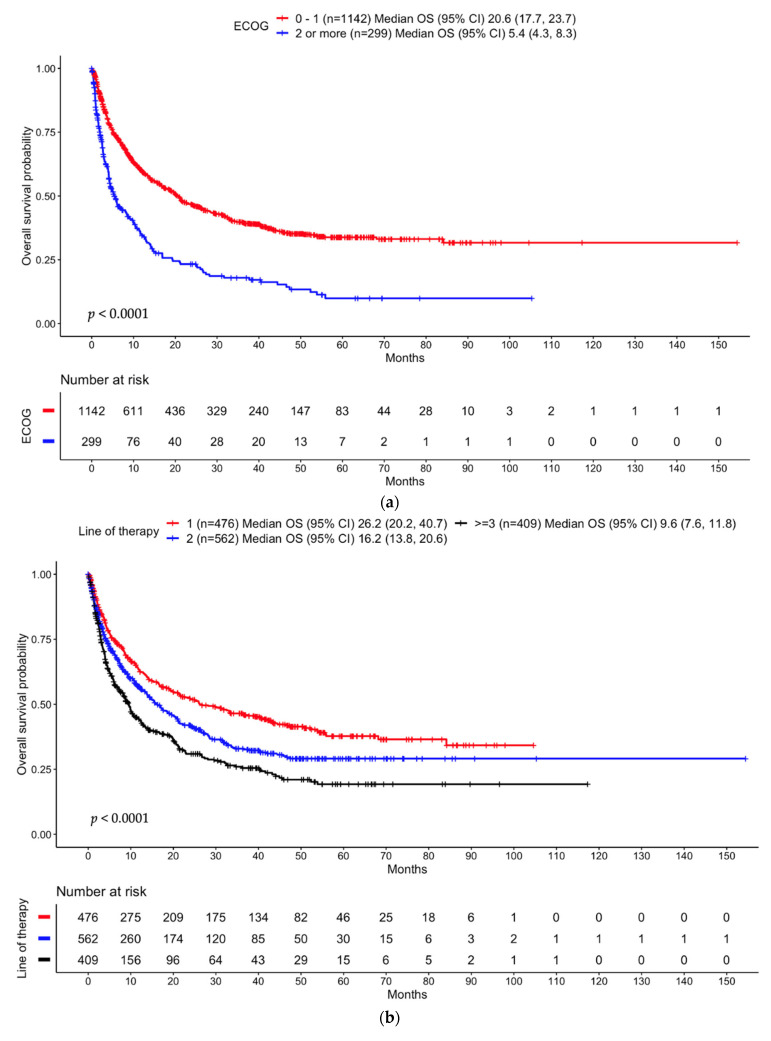

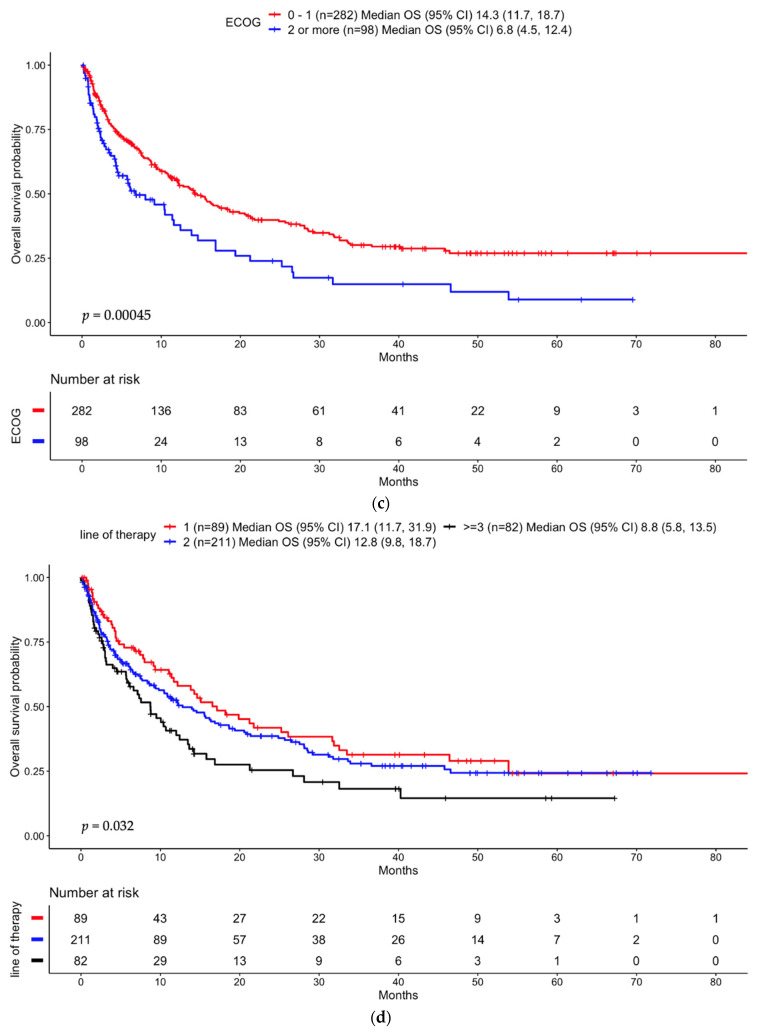
Overall survival probability by ECOG PS and lines of therapy in the entire cohort and anti-PD-(L)1 monotherapy NSCLC cohort: (**a**) ECOG PS 0–1 vs. ECOG PS ≥ 2 in the entire cohort; (**b**) Lines of therapy in the entire cohort; (**c**) ECOG PS 0–1 vs. ECOG PS ≥ 2 in the anti-PD-(L)1 monotherapy NSCLC cohort; (**d**) Lines of therapy in the anti-PD-(L)1 monotherapy NSCLC cohort.

**Figure 2:**
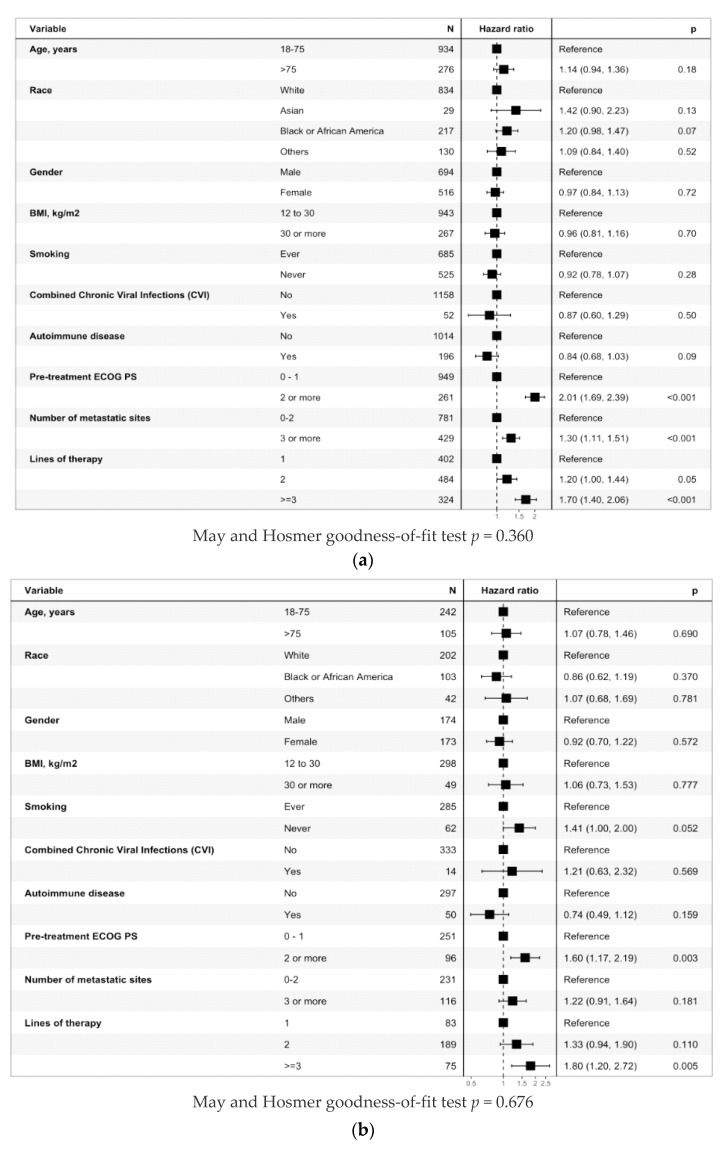
Overall survival hazard ratio by unique patient groups of interest in the: (**a**) entire cohort; (**b**) anti-PD-(L)1 monotherapy NSCLC cohort.

The median OS was 16.2 months (95% CI 13.8, 19.3). The median OS for patients with ECOG PS < 2 vs. ECOG PS ≥ 2 was 20.6 (95% CI 17.7, 23.7) vs. 5.4 (95% CI 4.3, 8.3) months, respectively, *p* < 0.001 (Figure 1a). In the multivariable analysis, ECOG PS ≥ 2 was associated with poor OS (HR = 2.01, *p* < 0.001) (Figure 2a). The previous number of LOTs also influenced OS, including the median OS of 26.2 months (95% CI 20.2, 40.7) for one prior LOT, 16.2 months (95% CI 13.8, 20.6) for 2 LOTs, and 9.6 months (95% CI 7.6, 11.8) for ≥3 LOTs (*p* < 0.001, Figure 1b). Race also influenced OS: median OS of 18.4 months (95% CI 14.7, 21.1) for White patients, 14.7 months (95% CI 8.5, 28.4) for others, 12 months (95% CI 8.8, 15.8) for Black patients, and 8.7 months (95% CI 6, 28.4) for Asian patients (*p* = 0.013, Supplementary Figure S3B).

#### 3.3.2. Anti-PD-(L)1 Monotherapy Non-Small Cell Lung Cancer Cohort

The median OS was 12.2 months (95% CI, 10.4, 15.7). ECOG PS, prior LOTs, history of AIDs, and smoking history significantly influenced OS. Median OS was 14.3 months in ECOG PS < 2 vs. 6.8 months in ECOG PS ≥ 2 (*p* < 0.001, Figure 1c). Again, the multivariable analysis noted that ECOG PS ≥ 2 was associated with poor OS (HR = 1.60, *p* = 0.003, Figure 2b). The previous number of LOTs also correlated negatively with OS, including the median OS of 17.1 months for one prior LOT, 12.8 months for two LOTs, and 8.8 months for ≥three LOTs (*p* = 0.032, Figure 1d). In addition, a history of AIDs (*p* = 0.044, Supplementary Figure S5D) and a history of smoking positively (*p* = 0.0029, Supplementary Figure S5F) influenced OS. In White patients, multivariable analysis showed that ECOG PS ≥ 2 was associated with poor OS (HR = 1.60, *p* = 0.003) as well as ≥three LOTs (HR = 1.91, *p* = 0.015) (Supplementary Figure S6). In Black patients, multivariable analysis showed that those who were never smokers had a poorer OS (HR = 2.00, *p* = 0.04, Supplementary Figure S7). Results for the entire NSCLC cohort can be found in the Supplementary Figures S2, S4 and S14.

### 3.4. Time to Treatment Failure (TTF)

#### 3.4.1. Entire Cohort

The median TTF was 4.53 months (95% CI 4.23, 5.1). ECOG PS, prior LOTs, number of metastatic sites, and BMI significantly influenced the TTF. The median TTF for patients with ECOG PS < 2 vs. ECOG PS ≥ 2 was 5.4 vs. 2.7 months, respectively (*p* < 0.001, Supplementary Figure S8A). The previous number of LOTs also influenced TTF, including the median TTF of 6.1 months for one prior LOT, 4.8 months for two LOTs, and 3.2 months for ≥three LOTs (*p* < 0.001) (Supplementary Figure S8D). In addition, BMI ≥30 mg/kg^2^ (*p* = 0.039, Supplement Figure S9E) and ≥three metastatic sites (*p* = 0.004, Supplementary Figure S9H) influenced TTF.

#### 3.4.2. Anti-PD-(L)1 Monotherapy Non-Small Cell Lung Cancer Cohort

The median TTF was 4.2 months (95% CI 3.27, 4.63). ECOG PS, prior LOTs, history of AIDs, and smoking history significantly influenced the TTF. The median TTF was 4.4 months in ECOG PS < 2 vs. 2.9 months in ECOG PS ≥ 2 (*p* < 0.001, Supplementary Figure S8C). The previous number of LOTs also influenced TTF, including the median TTF of 4.7 months for one prior LOT, 4.3 months for two LOTs, and 2.1 months for ≥three LOTs (*p* < 0.001, Supplementary Figure S8F). In addition, a history of AIDs (*p* = 0.016, Supplementary Figure S11D) and smoking history (*p* = 0.001, Supplementary Figure S11F) positively influenced TTF. In White patients, multivariable analysis showed that female gender (OR = 0.71, *p* = 0.028), ECOG PS ≥ 2 (OR = 1.60, *p* = 0.005), and ≥3 LOTs (OR = 1.81, *p* = 0.009) influenced TTF (Supplementary Figure S12). In Black patients, multivariable analysis showed that ≥three LOTs (OR = 2.63, *p* = 0.008) influenced TTF (Supplementary Figure S13). Results for the entire NSCLC cohort can be found in the Supplementary Figure S10.

## 4. Discussion

We present a large real-world evidence study evaluating ICI efficacy and safety across multiple tumor types and diverse patient populations. This is one of the most extensive real-world studies evaluating the ICI outcomes in patient populations typically underrepresented in registrational clinical trials. In our study, the overall incidence of any grade irAEs was 39.3%, and grade ≥ 3 was 12.6%. Our findings were similar to a previously reported sizeable retrospective study (n = 928) by Nebhan et al. [16], where they noted any grade irAEs was 41.3%, and grade ≥ 3 was 12.2%. In addition, similar results were also noted in a multicenter study (n = 976) [21], which noted any grade irAEs of 40.3%. We observed a median OS for the anti-PD-(L)1 monotherapy NSCLC cohort of 12.2 months which was similar to the median OS of 10.9 months for the NSCLC cohort in the Nebhan study [16]. The similar findings between the above multicenter, real-world retrospective studies increase the validity of the outcomes reported from our retrospective database.

For elderly patients (age > 75 years), we did not observe any difference in ICI efficacy or safety compared to younger patients in the entire patient population. These findings were confirmed in a less confounded anti-PD-(L)1 monotherapy-treated NSCLC cohort (OS: *p* = 0.69, TTF: *p* = 0.58, any grade irAEs: *p* = 0.097). Our findings are like those of the CheckMate 171 [12] study, where the median OS was similar in the entire cohort to those aged ≥70 or ≥75 years: 10.0 months, 10.0 months, and 11.2 months, respectively. Similar findings were noted again in a network metanalysis by Kim et al. [37]. Taken together, these results suggest that ICI therapy is safe and effective in elderly patients. For patients with ECOG PS ≥ 2, we noted poor survival but decreased incidence of irAE. In the anti-PD-(L)1 monotherapy NSCLC cohort, the median OS was 14.3 months in patients with ECOG PS < 2 vs. 6.8 months in those with ECOG PS ≥ 2 (*p* < 0.001). Similar results were noted in the forest plot analysis HR = 1.60 (*p* = 0.003). Our findings align with findings from CheckMate 153 and 171 studies [11,12] and a metanalysis by Tomasik et al. [17]. Therefore, we conclude that ECOG PS ≥ 2 strongly predicts poor ICI efficacy. PS is dependent on disease burden and/or other comorbidities. A study by Facchinetti et al. [38] noted that poor PS due to disease burden was associated with worse outcomes than poor PS due to other comorbidities (OS 2.8 vs. 11.8 months, respectively). Thus, determining the underlying reason for poor PS may aid clinical decision-making regarding the use of ICIs.

We noted that Black or Asian patients were less likely to develop any grade irAEs. In the anti-PD-(L)1 monotherapy NSCLC Cohort, the multivariable analysis noted Black patients were less likely to develop any-grade irAEs (OR 0.53, *p* = 0.023) compared to Whites, although, interestingly, no difference in ICI efficacy was observed (HR 0.86, *p* = 0.37). Our efficacy findings mirror those of Nazha et al. [18], where they did not find any difference in OS or PFS between Black and White patients. This suggests that patients’ self-identified race does not influence ICI efficacy. However, in contrast to our study, Nazha et al. noted a similar incidence of any-grade irAEs between cohorts. This could be due to a lower incidence of irAEs (28% vs. 41%) and a smaller sample size (n = 257 vs. 384) for Nazha et al. compared to ours; thus, their study may have been underpowered to identify a significant difference.

We did not find any difference in ICI efficacy or toxicity in patients with chronic viral infections. In the anti-PD-(L)1 monotherapy NSCLC cohort, similar results were noted for ICI efficacy but a lower incidence of any grade irAEs, although the sample size for the patients with chronic viral infections was small. Our findings were similar to the meta-analysis by Cook et al. [33] and a systematic review [39]. Overall, ICI therapy appears safe and effective in patients living with chronic viral infections.

A history of AIDs was not associated with improved ICI efficacy in the entire cohort or anti-PD-(L)1 monotherapy NSCLC Cohort (*p* = 0.09, *p* = 0.159) compared to patients without a history of AID. In the anti-PD-(L)1 monotherapy NSCLC cohort, the multivariate analysis did note an increased risk of irAEs in those with a history of AIDs (OR 1.93, *p* = 0.037). These results are similar to previously reported studies suggesting similar efficacy of ICI in patients with or without AIDs [27,28]. We found no association between sex or smoking status and ICI efficacy. Although, we noted a trend toward improved survival among patients with NSCLC and a smoking history. In addition, in the entire cohort, we noted that BMI >30 mg/kg^2^ was associated with an increased risk of any grade irAEs, but the signal was lost in the anti-PD-(L)1 monotherapy NSCLC cohort. Obesity remains a controversial issue; however, findings from our study do not support obesity as a predictive marker of ICI outcomes (either efficacy or toxicity), especially for NSCLC patients [21,22,23,24].

The line of therapy played a significant role in ICI efficacy, with an increased number of lines of therapy being associated with poor OS. These findings are similar to the previously reported study by Cortellini et al. [21] and the recently reported randomized DREAMseq trial [40], which suggest that ICIs may be most effective if administered in the first line setting.

Our study has several strengths and weaknesses. First, the diverse patient population includes multiple communities and academic cancer centers, aligning real-world evidence with standard oncology practices. In addition, given multiple confounding factors that are inherent in results for the entire cohort, we performed an analysis in a more uniform anti-PD-(L)1 monotherapy NSCLC cohort. Still, given the study’s retrospective nature, the results may be compromised by unknown factors such as lack of documentation. In addition, it should be noted that ethnicity, an important diverse and unique sub-population, and individual CVIs (e.g., HIV, HBV, and HCV) were not analyzed in our study due to limited cohort size. Furthermore, the subset analyses based on race and individual CVIs are limited due to our small sample size. Another limiting factor is the lack of data regarding prior lines of treatment and PD-(L)1 percentage scores, as we did not have this information available and, henceforth, were not able to perform a subset analysis.

## 5. Conclusions

We present one of the largest and most comprehensive studies evaluating the impact of ICI among underrepresented and unique patient populations. Overall, we observed that ICI was safe in the elderly population, patients living with a chronic viral infection, patients with poor performance status, and patients with high BMI. In addition, overall similar ICI results were noted regardless of the patient’s age, sex, BMI, smoking status, or history of chronic viral infections. We noted poor ECOG PS and an increased prior LOTs were associated with poor ICI efficacy, while a prior history of AIDs was associated with an increased risk of irAEs, and poor ECOG PS and Black race were associated with fewer irAEs. Findings from our study establish that ICI treatment is clinically effective and safe in these unique and typically underrepresented patient populations and, therefore, supports the use of ICI treatment in diverse, real-world patients.

## Figures and Tables

**Table 1:** Baseline characteristics.

Characteristics	Entire Cohort N = 1453 *n* (%)	PD-(L)1 Monotherapy NSCLC Cohort N = 384 *n* (%)
**Age, median (IQR) years**	65.8 (56.6, 74.3)	70.1 (61.8, 76.3)
18–75	1118 (77.2)	264 (68.9)
>75	330 (22.8)	119 (31.1)
**Race**		
Asian	39 (2.7)	13 (3.4)
Black	237 (16.3)	111 (28.9)
White ^a^	1012 (69.6)	227 (59.1)
Others	165 (11.4)	33 (8.6)
**Gender**		
Male	838 (57.8)	195 (50.8)
Female	612 (42.2)	189 (49.2)
**BMI, kg/m^2^**		
12 ≤ BMI < 30	1110 (77.9)	322 (85)
BMI ≥ 30	315 (22.1)	57 (15)
**Smoking Status**		
Ever Smoker ^b^	817 (56.5)	319 (83.1)
Never Smoker	628 (43.5)	65 (16.9)
**Chronic Viral Infections (CVI)**		
Combined CVI ^c^	68 (4.7)	16 (4.2)
Hepatitis B (HBV)	25 (1.7)	3 (0.8)
Hepatitis C (HCV)	32 (2.2)	6 (1.6)
HIV	18 (1.2)	8 (2.1)
**History of AID ^d^**	228 (15.7)	54 (14.1)
**Pre-treatment ECOG PS**		
0	383 (26.5)	78 (20.4)
1	761 (52.7)	205 (53.7)
≥2	300 (20.8)	99 (25.9)
**ICIs**		
Atezolizumab	47 (3.2)	26 (6.8)
Avelumab	3 (0.2)	1 (0.3)
Durvalumab	17 (1.2)	9 (2.3)
Ipilimumab	163 (11.2)	-
Nivolumab	539 (37.1)	245 (63.8)
Nivolumab + ipilimumab	192 (13.2)	-
Pembrolizumab	323 (22.2)	103 (26.8)
Pembrolizumab + ipilimumab	14 (1)	-
IO plus chemo	38 (2.6)	-
Others ^e^	117 (8.1)	-
**Cancer types**		
Lung cancer	499 (34.4) ^f^	384 (100)
Adenocarcinoma	312 (62.5)	256 (66.7)
Squamous	116 (23.2)	105 (27.3)
Others	69 (13.8)	-
Melanoma	403 (27.8) ^g^	-
Cutaneous	293 (72.7)	-
Others	75 (18.6)	-
GI cancers	104 (7.2)	-
Kidney cancers	100 (6.9)	-
Others	346 (23.8)	-

AID = autoimmune disease; BMI = body mass index; ECOG = Eastern Cooperative Oncology Group; HIV = human immunodeficiency virus; ICI = immune checkpoint inhibitors; NSCLC = non-small cell lung cancer; PS = performance status. ^a^ White patients include non-Hispanic and Hispanic Whites, as ethnicity data were not available. ^b^ Ever smoker = patients with active or previous/occasional smoking history. ^c^ Combined CVI = history of any of the following: human immunodeficiency virus (HIV), hepatitis B virus (HBV), or hepatitis C virus (HCV) infection. ^d^ AIDs included a diagnosis of a variety of autoimmune conditions, such as Hashimoto’s disease, primary biliary cirrhosis, hypothyroidism, pyoderma gangrenosum, and thyrotoxicosis (see Supplementary Materials for complete definition of AIDs). ^e^ Others = included interleukin 2, pembrolizumab plus axitinib, and undefined (n = 132). ^f^ Total patients with NSCLC. Sum of subtypes of NSCLC exceeds the total number of patients with NSCLC, as some patients had both adenocarcinoma and squamous cell histology. ^g^ Total patients with melanoma that received ICIs as adjuvant therapy and those with metastatic disease.

**Table 2:** Multivariate analysis of immune-related adverse events (irAEs) in entire cohort.

Characteristic	Entire Cohort			
	Any Grade irAEs OR (95% CI)	*p*-Value	Grade ≥ 3 irAEs OR (95% CI)	*p*-Value
**Age, years**		0.446		0.425
18–75	*ref*		*ref*	
>75	1.11 (0.85, 1.43)		0.85 (0.57, 1.25)	
**Race**				
Asian	0.43 (0.19, 0.90)	0.033	0.56 (0.13, 1.60)	0.344
Black	0.54 (0.39, 0.73)	<0.001	0.49 (0.28, 0.81)	0.008
White	*ref*	-	*ref*	-
Other	0.81 (0.57, 1.14)	0.233	0.79 (0.46, 1.3)	0.383
**Gender**		0.356		0.439
Male	*ref*		*ref*	
Female	1.11 (0.89, 1.38)		0.88 (0.63, 1.21)	
**BMI, kg/m^2^**		<0.001		0.001
12 ≤ BMI < 30	*ref*		*ref*	
BMI ≥ 30	1.44 (1.11, 1.86)		1.61 (1.13, 2.28)	
**ECOG PS**		<0.001		0.001
0–1	*ref*		*ref*	
≥2	0.46 (0.34, 0.62)		0.45 (0.27, 0.71)	

BMI = body mass index; CI = confidence interval; ECOG = Eastern Cooperative Oncology Group; irAE = immune-related adverse event; OR = odds ratio; PS = performance status. Omnibus goodness-of-fit test *p* = 0.110 for any grade irAEs. Omnibus goodness-of-fit test *p* = 0.186 for grade ≥ 3 irAEs.

**Table 3:** Multivariate analysis of immune-related adverse events (irAEs) in anti-PD-(L)1 monotherapy NSCLC patients.

Characteristic	Anti-PD-(L)1 NSCLC Cohort			
	Any Grade irAEs OR (95% CI)	*p*-Value	Grade ≥ 3 irAEs OR (95% CI)	*p*-Value
**Age, years**		0.097		0.093
18–75	*ref*		*ref*	
>75	1.52 (0.93, 2.47)		1.98 (0.89, 4.42)	
**Race**				
Black	0.53 (0.30, 0.90)	0.023	0.44 (0.14, 1.16)	0.123
White	*ref*	-	*ref*	-
Other	0.56 (0.25, 1.15)	0.128	0.70 (0.16, 2.21)	0.583
**Gender**		0.136		0.292
Male	*ref*		*ref*	
Female	1.43 (0.89, 2.28)		1.53 (0.70, 3.50)	
**BMI, kg/m^2^**	-	-		0.252
12 ≤ BMI < 30			*ref*	
BMI ≥ 30			1.72 (0.64, 4.17)	
**Combined CVI ^a^**		0.006		0.041
Yes	*ref*		*ref*	
No	0.22 (0.07, 0.64)		0.23 (0.06, 1.12)	
**History of AID ^b^**		0.037		0.293
Yes	1.93 (1.03, 3.59)		1.65 (0.61, 4.00)	
No	*ref*		*ref*	
**ECOG PS**		0.036	-	-
0–1	*ref*			
≥2	0.55 (0.31, 0.95)			

AID = autoimmune disease; BMI = body mass index; CI = confidence interval; CVI = chronic viral infections; ECOG = Eastern Cooperative Oncology Group; irAE = immune-related adverse event; NSCLC = non-small cell lung cancer; OR = odds ratio; PS = performance status. ^a^ Combined CVI = history of any of the following: human immunodeficiency virus (HIV), hepatitis B virus (HBV), or hepatitis C virus (HCV) infection. ^b^ AIDs included a diagnosis of a variety of autoimmune conditions, such as Hashimoto’s disease, primary biliary cirrhosis, hypothyroidism, pyoderma gangrenosum, and thyrotoxicosis (see Supplementary Materials for complete definition of AID). Omnibus goodness-of-fit test *p* = 0.892 for any grade irAEs. Omnibus goodness-of-fit test *p* = 0.424 for grade ≥ 3 irAEs.

## Data Availability

The data presented in this study are available on request from the corresponding author. The research dataset has removed mode, but not all protected health information (for example, actual dates of immune checkpoint inhibitor treatment were included as needed for analysis but would be considered PFI). The dataset is also considered property of Georgetown University Medical Center and not owned by the investigators. However, upon request, we are willing to share portions of the data for appropriate review. Requests can be made through the corresponding author (Neil J. Shah; email: shahn6@mskcc.org).

## Supplementary Materials

The following supporting information can be downloaded at https://www.mdpi.com/article/10.3390/cancers16122223/s1, Text S1: Definition of autoimmune disease (AID); Figure S1: Flow diagram of patient cohorts; Table S1: Baseline characteristics for the entire NSCLC cohort; Table S2: Univariate analysis of immune-related adverse events (irAEs) in the entire cohort; Table S3: Univariate analysis of immune-related adverse events (irAEs) in the entire NSCLC cohort; Table S4: Multivariate analysis of immune-related adverse events (irAEs) in the entire NSCLC cohort; Table S5: Univariate analysis of immune-related adverse events (irAEs) in PD-L1 monotherapy NSCLC patients; Table S6: Multivariate analysis of immune-related adverse events (irAEs) in White patients in the anti-PD-(L)1 monotherapy NSCLC cohort; Table S7: Multivariate analysis of immune-related adverse events (irAEs) in Black patients in the anti-PD-(L)1 monotherapy NSCLC cohort; Figure S2: Overall survival probability by ECOG PS and lines of therapy in the entire NSCLC cohort; Figure S3: Overall survival probability in all patients treated with ICIs by unique cohorts; Figure S4: Overall survival probability in NSCLC patients treated with ICIs by unique cohorts; Figure S5: Overall survival probability in NSCLC patients treated with PD-(L)1 monotherapy by unique cohorts; Figure S6: Overall survival hazard ratio by unique patient groups of interest in White patients in the anti-PD-(L)1 monotherapy NSCLC cohort; Figure S7: Overall survival hazard ratio by unique patient groups of interest in Black patients in the anti-PD-(L)1 monotherapy NSCLC cohort; Figure S8: Time to treatment failure (TTF) probability by ECOG performance status and lines of therapy in the entire cohort, entire NSCLC cohort, and PD-(L)1 monotherapy cohort; Figure S9: Time to treatment failure (TTF) probability in patients treated with ICIs by unique cohorts; Figure S10: Time to treatment failure (TTF) probability in NSCLC patients treated with ICIs by unique cohorts; Figure S11: Time to treatment failure (TTF) probability in NSCLC patients treated with PD-L1 monotherapy by unique cohorts; Figure S12: Time to treatment failure hazard ratio by unique patient groups of interest in White patients in the anti-PD-(L)1 monotherapy NSCLC cohort; Figure S13: Time to treatment failure hazard ratio by unique patient groups of interest in Black patients in the anti-PD-(L)1 monotherapy NSCLC cohort; Figure S14: Overall survival hazard ratio in the entire NSCLC cohort treated with ICIs by unique cohorts.
