# Supplementary material for: Clinical Outcomes of Immune Checkpoint Inhibitors in Unique Cohorts Underrepresented in Clinical Trials
Source: Cancers (Basel). 2024 Jun 14;16(12):2223. doi: 10.3390/cancers16122223 (PMC11202168; doi:10.3390/cancers16122223)
Supplement: Supplementary file 1 [file cancers-16-02223-s001.zip › cancers-2914790-supplementary.pdf]

Supplementary Material

Text S1. Definition of autoimmune disease (AID).

Figure S1. Flow diagram of patient cohorts.

Table S1. Baseline characteristics for the entire NSCLC cohort.

Table S2. Univariate analysis of immune-related adverse events (irAEs) in the entire cohort.

Table S3. Univariate analysis of immune-related adverse events (irAEs) in the entire NSCLC cohort.

Table S4. Multivariate analysis of immune-related adverse events (irAEs) in the entire NSCLC cohort.

Table S5. Univariate analysis of immune-related adverse events (irAEs) in PD-L1 monotherapy NSCLC patients.

Table S6. Multivariate analysis of immune-related adverse events (irAEs) in White patients in the anti-PD-(L)1 monotherapy NSCLC cohort.

Table S7. Multivariate analysis of immune-related adverse events (irAEs) in Black patients in the anti-PD-(L)1 monotherapy NSCLC cohort.

Figure S2. Overall survival probability by ECOG PS and lines of therapy in the entire NSCLC cohort.

Figure S3. Overall survival probability in all patients treated with ICIs by unique cohorts.

Figure S4. Overall survival probability in NSCLC patients treated with ICIs by unique cohorts.

Figure S5. Overall survival probability in NSCLC patients treated with PD-(L)1 monotherapy by unique cohorts.

Figure S6. Overall survival hazard ratio by unique patient groups of interest in White patients in the anti-PD-(L)1 monotherapy NSCLC cohort.

Figure S7. Overall survival hazard ratio by unique patient groups of interest in Black patients in the anti-PD-(L)1 monotherapy NSCLC cohort.

Figure S8. Time to treatment failure (TTF) probability by ECOG performance status and lines of therapy in the entire cohort, entire NSCLC cohort, and PD-(L)1 monotherapy cohort.

Figure S9. Time to treatment failure (TTF) probability in patients treated with ICIs by unique cohorts.

Figure S10. Time to treatment failure (TTF) probability in NSCLC patients treated with ICIs by unique cohorts.

Figure S11. Time to treatment failure (TTF) probability in NSCLC patients treated with PD-L1 monotherapy by unique cohorts.

Figure S12. Time to treatment failure hazard ratio by unique patient groups of interest in White patients in the anti-PD-(L)1 monotherapy NSCLC cohort.

Figure S13. Time to treatment failure hazard ratio by unique patient groups of interest in Black patients in the anti-PD-(L)1 monotherapy NSCLC cohort.

Figure S14. Overall survival hazard ratio in the entire NSCLC cohort treated with ICIs by unique cohorts.

Text S1. Definition of autoimmune disease (AID).

Autoimmune disease (AID) was defined as a diagnosis of any of the following conditions: Addison's disease, alopecia areata, ankylosing spondylitis, antiphospholipid, autoimmune hemolytic anemia, autoimmune hepatitis, autoimmune not otherwise specified, celiac disease, Crohn’s disease, dermatopolymyositis, eczema, giant cell arteritis, Guillain-Barre syndrome, Hashimoto’s disease, hypothyroidism, hyperthyroidism, idiopathic thrombocytopenic purpura, iridocyclitis, Meniere disease, multiple sclerosis, myasthenia gravis, pernicious anemia, pemphigus, polyarteritis nodosa, polymyalgia rheumatica, primary biliary cirrhosis, psoriasis, pyoderma gangrenosum, rheumatoid arthritis, sarcoidosis, scleroderma, sicca syndrome, sweet’s syndrome, Sjogren’s syndrome, systemic lupus erythematosus, systemic sclerosis, thyrotoxicosis, type 1 diabetes mellitus, ulcerative colitis, vitiligo, Wegener granulomatosis. Patients with a history of any of the aforementioned conditions were included in the AID subgroup, but it is unknown if AID was active, or if patients were actively receiving immunomodulators, at the time of ICI treatment. For hypothyroidism, we were unable to differentiate if this condition was surgically-induced.

Figure S1. Flow diagram of patient cohorts.

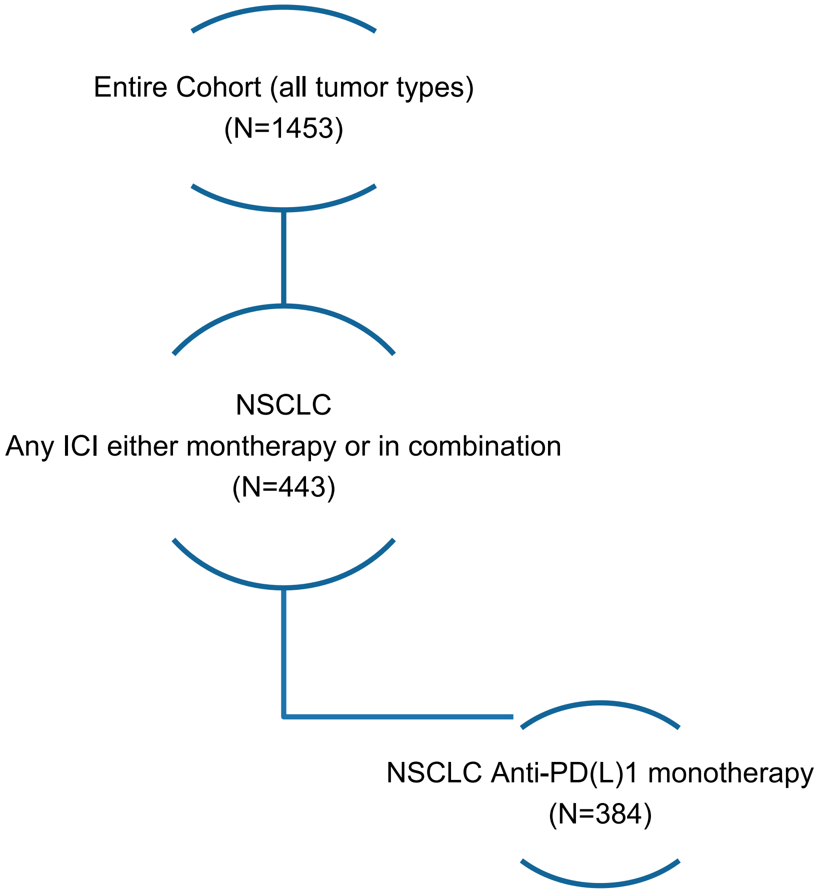

Table S1. Baseline characteristics for the entire NSCLC cohort.

Characteristics	Entire NSCLC cohort<br>N=443<br>*n* (%)
**Age - Median (IQR), years**	68.83 (60.84,75.72)
18 – 75	316 (71.5)
> 75	126 (28.5)
**Race**	
Asian	14 (3.2)
Black	126 (28.4)
White	267 (60.3)
Others	36 (8.1)
**Gender**	
Male	224 (50.6)
Female	219 (49.4)
**Race by gender (male)**	
Asian	8 (2.1)
Black	73 (19.0)
White	122 (31.8)
Others	21 (5.5)
**BMI, kg/m^2**	
12≤BMI<30	370 (85.1)
BMI≥30	65 (14.9)
**Smoking Status**	
Ever Smoker[a]	369 (83.3)
Never Smoker	74 (16.7)
**Chronic Viral Infections (CVI)**	
Combined CVI[b]	20 (4.5)
Hepatitis B (HBV)	4 (0.9)
Hepatitis C (HCV)	9 (2)
HIV	9 (2)
**History of AID[c]**	62 (14)
**Pre-treatment ECOG PS**	
0	101 (23)
1	235 (53.5)
≥2	103 (23.5)
**ICIs**	
Atezolizumab	26 (5.9)
Avelumab	1 (0.2)
Durvalumab	9 (2)
Ipilimumab	1 (0.2)
Nivolumab	245 (55.3)
Nivolumab + Ipilimumab	2 (0.5)

Pembrolizumab	103 (23.3)
Pembrolizumab + Ipilimumab	1 (0.2)
IO plus chemo	37 (8.4)
Others[d]	18 (4.1)
**Cancer types**	
Lung Cancer	443 (100)[e]
Adenocarcinoma	306 (69.1)
Squamous	112 (25.3)
Others	-
Melanoma	-
Cutaneous	-
Others	-
GI Cancers	-
Kidney Cancers	-
Others	-

NSCLC=non-small cell lung cancer; BMI=body mass index; HIV=human immunodeficiency virus; AID=autoimmune disease; ECOG=Eastern Cooperative Oncology Group; PS=performance status; ICI=immune checkpoint inhibitors

[a]Ever smoker=patients with active or previous/occasional smoking history
[b]Combined CVI=history of any of the following: human immunodeficiency virus (HIV), hepatitis B virus (HBV), or hepatitis C virus (HCV) infection
[c]AID=included a diagnosis of a variety of autoimmune conditions, such as Hashimoto's disease, primary biliary cirrhosis, hypothyroidism, pyoderma gangrenosum, and thyrotoxicosis (see Supplement for complete definition of AID)
[d]Others=included interleukin 2, pembrolizumab plus axitinib, and undefined (n=132)
[e]Total patients with NSCLC. Sum of subtypes of NSCLC exceeds the total number of patients with NSCLC as some patients had both adenocarcinoma and squamous cell histology.

Safety analysis.

Table S2. Univariate analysis of immune-related adverse events (irAEs) in the entire cohort.

Characteristic	Entire cohort					
	**Any grade irAEs N=569 *n* (%)**	**No irAEs N=879 *n* (%)**	***p-value***	**Grade ≥3 irAEs N=183 *n* (%)**	**No Grade ≥3 irAEs N=1,265 *n* (%)**	***p-value***
**Age, years**			0.315			0.413
18 – 75	429 (39)	684 (61)		146 (13)	967 (87)	
> 75	138 (42)	192 (58)		37 (11)	293 (89)	
**Race**			<0.001			0.021
Asian	10 (26)	29 (74)		3 (7.7)	36 (92)	
Black	65 (28)	171 (72)		17 (7.2)	219 (93)	
White	432 (43)	577 (57)		143 (14)	866 (86)	
Others	62 (38)	102 (62)		20 (12)	144 (88)	
**Gender**			0.564			0.357
Male	323 (39)	512 (61)		112 (13)	723 (87)	
Female	246 (40)	364 (60)		71 (12)	539 (88)	
**BMI, kg/m^2**			0.004			0.004
12≤BMI<30	415 (38)	691 (62)		126 (11)	980 (89)	
BMI ≥30	147 (47)	167 (53)		56 (18)	258 (82)	
**Smoking Status**			0.757			0.741
Ever Smoker[a]	317 (39)	498 (61)		101 (12)	714 (88)	
Never Smoker	249 (40)	376 (60)		82 (13)	543 (87)	
**Combined CVI[b]**			0.358			0.413
Yes	30 (45)	36 (55)		11 (17)	55 (83)	
No	539 (39)	843 (61)		172 (12)	1,210 (88)	
**History of AID[c]**			0.219			0.860
Yes	98 (43)	129 (57)		30 (13)	197 (87)	
No	471 (39)	750 (61)		153 (13)	1,068 (87)	
**ECOG PS**			<0.001			0.001
0-1	489 (43)	651 (57)		162 (14)	978 (86)	
≥2	78 (26)	222 (74)		20 (6.7)	280 (93)	

irAE=immune-related adverse event; BMI=body mass index; CVI=chronic viral infections; AID=autoimmune disease; ECOG=Eastern Cooperative Oncology Group; PS=performance status;

[a]Ever smoker=patients with active or previous/occasional smoking history
[b]Combined CVI=history of any of the following: human immunodeficiency virus (HIV), hepatitis B virus (HBV), or hepatitis C virus (HCV) infection
[c]AID=included a diagnosis of a variety of autoimmune conditions, such as Hashimoto's disease, primary biliary cirrhosis, hypothyroidism, pyoderma gangrenosum, and thyrotoxicosis (see Supplement for complete definition of AID)

Table S3. Univariate analysis of immune-related adverse events (irAEs) in the entire NSCLC cohort.

Characteristic	Entire NSCLC cohort					
	Any grade irAEs N=142 *n* (%)	No irAEs N=300 *n* (%)	*p-value*	Grade ≥3 irAEs N=40 *n* (%)	No Grade ≥3 irAEs N=402 *n* (%)	*p-value*
**Age, years**			0.037			0.063
18 – 75	91 (29)	224 (71)		23 (7.3)	292 (93)	
> 75	50 (40)	76 (60)		17 (13)	109 (87)	
**Race**			0.051			0.474
Black	31 (25)	95 (75)		8 (6.3)	118 (94)	
White	97 (36)	169 (64)		27 (10)	239 (90)	
Others	14 (28)	36 (72)		5 (10)	45 (90)	
**Gender**			0.006			0.114
Male	58 (26)	166 (74)		15 (6.7)	209 (93)	
Female	84 (39)	134 (61)		25 (11)	193 (89)	
**BMI, kg/m^2**			0.408			0.454
12≤BMI<30	116 (31)	254 (69)		32 (8.6)	338 (91)	
BMI ≥30	24 (38)	40 (62)		8 (12)	56 (88)	
**Smoking Status**			0.592			0.621
Ever Smoker[a]	121 (33)	248 (67)		35 (9.5)	334 (91)	
Never Smoker	21 (29)	52 (71)		5 (6.8)	68 (93)	
**Combined CVI[b]**			0.046			0.096
Yes	11 (55)	9 (45)		4 (20)	16 (80)	
No	131 (31)	291 (69)		36 (8.5)	386 (91)	
**History of AID[c]**			0.026			0.367
Yes	28 (45)	34 (55)		8 (13)	54 (87)	
No	114 (30)	266 (70)		32 (8.4)	348 (92)	
**ECOG PS**			0.010			0.146
0-1	119 (36)	216 (64)		34 (10)	301 (90)	
≥2	22 (21)	81 (79)		5 (4.9)	98 (95)	

irAE=immune-related adverse event; BMI=body mass index; CVI=chronic viral infections; AID=autoimmune disease; ECOG=Eastern Cooperative Oncology Group; PS=performance status

[a]Ever smoker=patients with active or previous/occasional smoking history
[b]Combined CVI=history of any of the following: human immunodeficiency virus (HIV), hepatitis B virus (HBV), or hepatitis C virus (HCV) infection
[c]AID=included a diagnosis of a variety of autoimmune conditions, such as Hashimoto's disease, primary biliary cirrhosis, hypothyroidism, pyoderma gangrenosum, and thyrotoxicosis (see Supplement for complete definition of AID)

Table S4. Multivariate analysis of immune-related adverse events (irAEs) in the entire NSCLC cohort.

Characteristic	Entire NSCLC cohort			
	**Any grade irAEs OR (95% CI)**	***p-value***	**Grade ≥3 irAEs OR (95% CI)**	***p-value***
**Age, years**		0.073		0.041
18 – 75	*ref*		*ref*	
> 75	1.53 (0.96, 2.42)		2.09 (1.02, 4.24)	
**Race**				
Black	0.58 (0.35, 0.96)	0.036	0.65 (0.26, 1.46)	0.318
White	*ref*	-	*ref*	-
Others	0.61 (0.29, 1.22)	0.174	0.76 (0.21, 2.12)	0.636
**Gender**		0.031		0.119
Male	*ref*		*ref*	
Female	1.60 (1.05, 2.46)		1.75 (0.88, 3.60)	
**Combined CVI[a]**		0.007		0.036
Yes	*ref*		*ref*	
No	0.26 (0.10, 0.69)		0.27 (0.09, 1.04)	
**History of AID[b]**		0.044	-	-
Yes	1.81 (1.01, 3.23)			
No	*ref*			
**ECOG PS**		0.008		0.099
0-1	*ref*		*ref*	
≥2	0.48 (0.28, 0.82)		0.44 (0.15, 1.07)	

irAE=immune-related adverse event; CVI=chronic viral infections; AID=autoimmune disease; ECOG=Eastern Cooperative Oncology Group; PS=performance status

[a]Combined CVI=history of any of the following: human immunodeficiency virus (HIV), hepatitis B virus (HBV), or hepatitis C virus (HCV) infection
[b]AID=included a diagnosis of a variety of autoimmune conditions, such as Hashimoto's disease, primary biliary cirrhosis, hypothyroidism, pyoderma gangrenosum, and thyrotoxicosis (see Supplement for complete definition of AID)

Omnibus goodness-of-fit test p = 0.665 for any grade irAEs. Omnibus goodness-of-fit test p = 0.106 for grade ≥3 irAEs.

Table S5. Univariate analysis of immune-related adverse events (irAEs) in PD-L1 monotherapy NSCLC patients.

Characteristic	PD-L1 monotherapy NSCLC cohort					
	**Any grade irAEs N=117 *n* (%)**	**No irAEs N=266 *n* (%)**	***p-value***	**Grade ≥3 irAEs N=31 *n* (%)**	**No Grade ≥3 irAEs N=352 *n* (%)**	***p-value***
**Age, years**			0.045			0.050
18 – 75	71 (27)	192 (73)		16 (6.1)	247 (94)	
> 75	45 (38)	74 (62)		15 (13)	104 (87)	
**Race**			0.026			0.186
Black	25 (23)	86 (77)		5 (4.5)	106 (95)	
White	81 (36)	145 (64)		23 (10)	203 (90)	
Others	11 (24)	35 (76)		3 (6.5)	43 (93)	
**Gender**			0.026			0.109
Male	49 (25)	146 (75)		11 (5.6)	184 (94)	
Female	68 (36)	120 (64)		20 (11)	168 (89)	
**BMI, kg/m^2**			0.467			0.195
12≤BMI<30	96 (30)	226 (70)		24 (7.5)	298 (93)	
BMI ≥30	20 (36)	36 (64)		7 (12)	49 (88)	
**Smoking Status**			0.755			>0.999
Ever Smoker[a]	99 (31)	220 (69)		26 (8.2)	293 (92)	
Never Smoker	18 (28)	46 (72)		5 (7.8)	59 (92)	
**Combined CVI[b]**			0.048			0.131
Yes	9 (56)	7 (44)		3 (19)	13 (81)	
No	108 (29)	259 (71)		28 (7.6)	339 (92)	
**History of AID[c]**			0.026			0.176
Yes	24 (44)	30 (56)		7 (13)	47 (87)	
No	93 (28)	236 (72)		24 (7.3)	305 (93)	
**ECOG PS**			0.045			0.275
0-1	95 (34)	187 (66)		26 (9.2)	256 (91)	
≥2	22 (22)	77 (78)		5 (5.1)	94 (95)	

irAE=immune-related adverse event; BMI=body mass index; CVI=chronic viral infections; AID=autoimmune disease; ECOG=Eastern Cooperative Oncology Group; PS=performance status;

[a]Ever smoker=patients with active or previous/occasional smoking history
[b]Combined CVI=history of any of the following: human immunodeficiency virus (HIV), hepatitis B virus (HBV), or hepatitis C virus (HCV) infection
[c]AID=included a diagnosis of a variety of autoimmune conditions, such as Hashimoto’s disease, primary biliary cirrhosis, hypothyroidism, pyoderma gangrenosum, and thyrotoxicosis (see Supplement for complete definition of AID)

Table S6. Multivariate analysis of immune-related adverse events (irAEs) in White patients in the anti-PD-(L)1 monotherapy NSCLC cohort.

**Characteristic**	**White patients in anti-PD-(L)1 monotherapy NSCLC cohort**			
	**Any grade irAEs OR (95% CI)**	***p-value***	**Grade ≥3 irAEs OR (95% CI)**	***p-value***
**Age, years**		0.124		0.190
18 – 75	*ref*		*ref*	
> 75	1.59 (0.88, 2.87)		1.83 (0.74, 4.55)	
**Gender**		0.215		0.172
Male	*ref*		*ref*	
Female	1.44 (0.81, 2.57)		1.96 (0.77, 5.48)	
**BMI, kg/m^2**	-	-		0.056
12≤BMI<30			*ref*	
BMI ≥30			2.63 (0.93, 6.94)	
**ECOG PS**		0.008	-	-
0-1	*ref*			
≥2	0.35 (0.16, 0.70)			

irAE=immune-related adverse event; BMI=body mass index; ECOG=Eastern Cooperative Oncology Group; PS=performance status

Omnibus goodness-of-fit test p = 0.756 for any grade irAEs. Omnibus goodness-of-fit test p = 0.981 for grade ≥3 irAEs.

Table S7. Multivariate analysis of immune-related adverse events (irAEs) in Black patients in the anti-PD-(L)1 monotherapy NSCLC cohort.

Characteristic	Black patients in anti-PD-(L)1 monotherapy NSCLC cohort			
	**Any grade irAEs OR (95% CI)**	***p-value***	**Grade ≥3 irAEs OR (95% CI)**	***p-value***
**Age, years**		0.059		0.800
18 – 75	*ref*		*ref*	
> 75	2.79 (0.94, 8.07)		1.40 (0.06, 17.4)	
**Gender**		0.085		0.759
Male	*ref*		*ref*	
Female	2.29 (0.90, 6.02)		1.40 (0.15, 14.4)	
**Combined CVI[a]**		0.028		0.010
Yes	*ref*		*ref*	
No	0.18 (0.04, 0.86)		0.03 (0.00, 0.40)	
**History of AID[b]**	-	-		0.060
Yes			*ref*	
No			12.4 (0.97, 31.0)	

irAE=immune-related adverse event; CVI=chronic viral infections; AID=autoimmune disease; ECOG=Eastern Cooperative Oncology Group; PS=performance status

[a]Combined CVI=history of any of the following: human immunodeficiency virus (HIV), hepatitis B virus (HBV), or hepatitis C virus (HCV) infection
[b]AID=included a diagnosis of a variety of autoimmune conditions, such as Hashimoto's disease, primary biliary cirrhosis, hypothyroidism, pyoderma gangrenosum, and thyrotoxicosis (see Supplement for complete definition of AID)

Omnibus goodness-of-fit test p = 0.575 for any grade irAEs. Omnibus goodness-of-fit test p = 0.228 for grade ≥3 irAEs.

Efficacy analysis.

Figure S2. Overall survival probability by ECOG PS and lines of therapy in the entire NSCLC cohort.

2A. ECOG PS 0-1 vs. ECOG PS ≥2 in the entire NSCLC cohort.

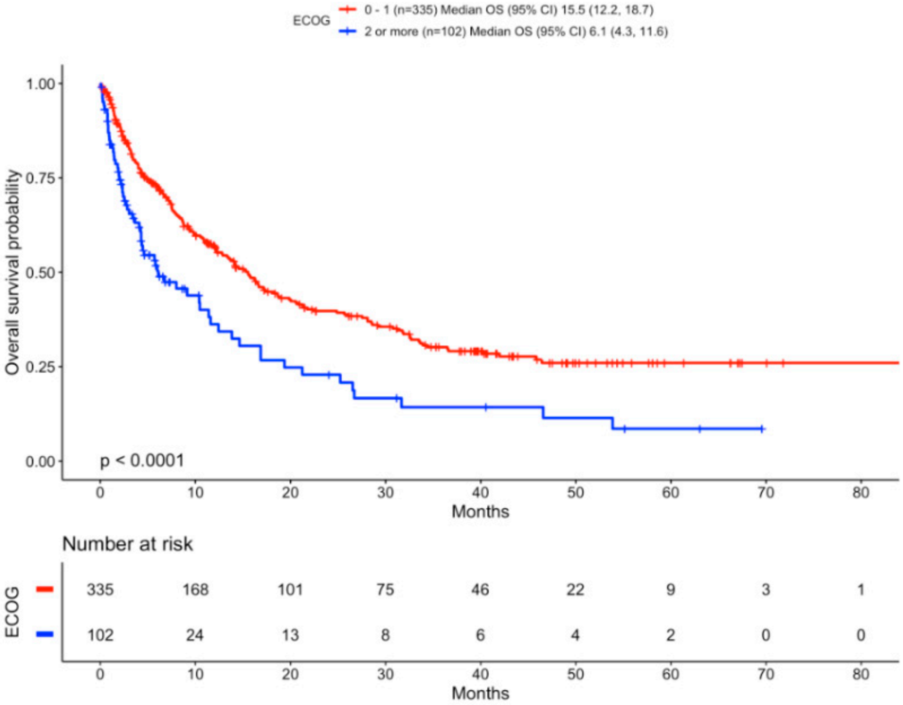

2B. Lines of therapy in the entire NSCLC cohort.

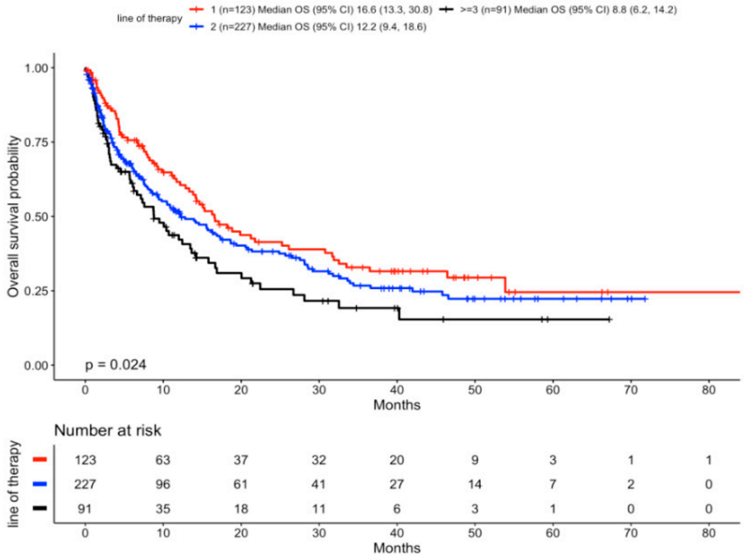

Figure S3. Overall survival probability in all patients treated with ICIs by unique cohorts.

A. Age ≤75 years vs. Age >75 years

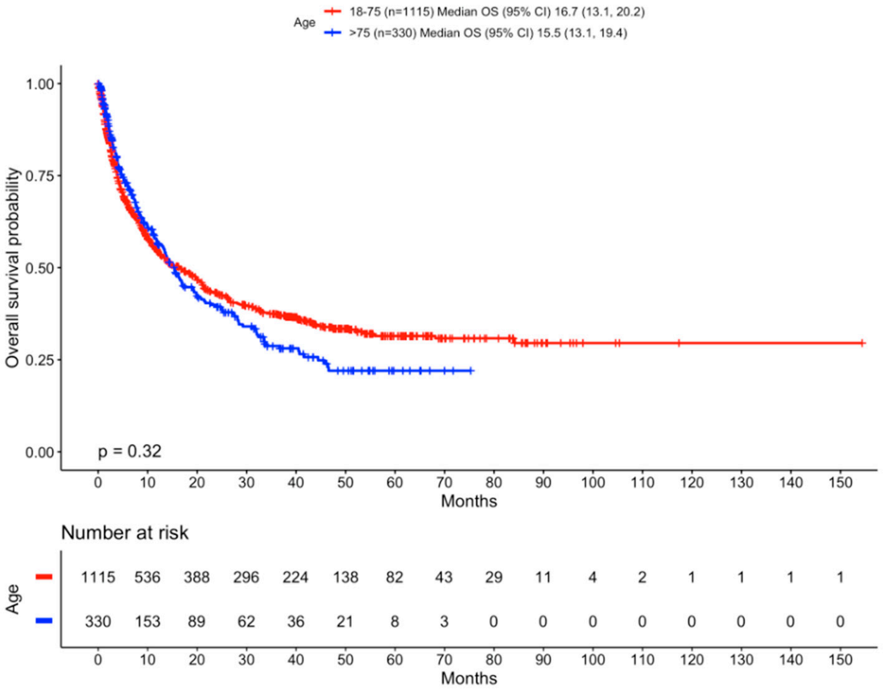

B. Race

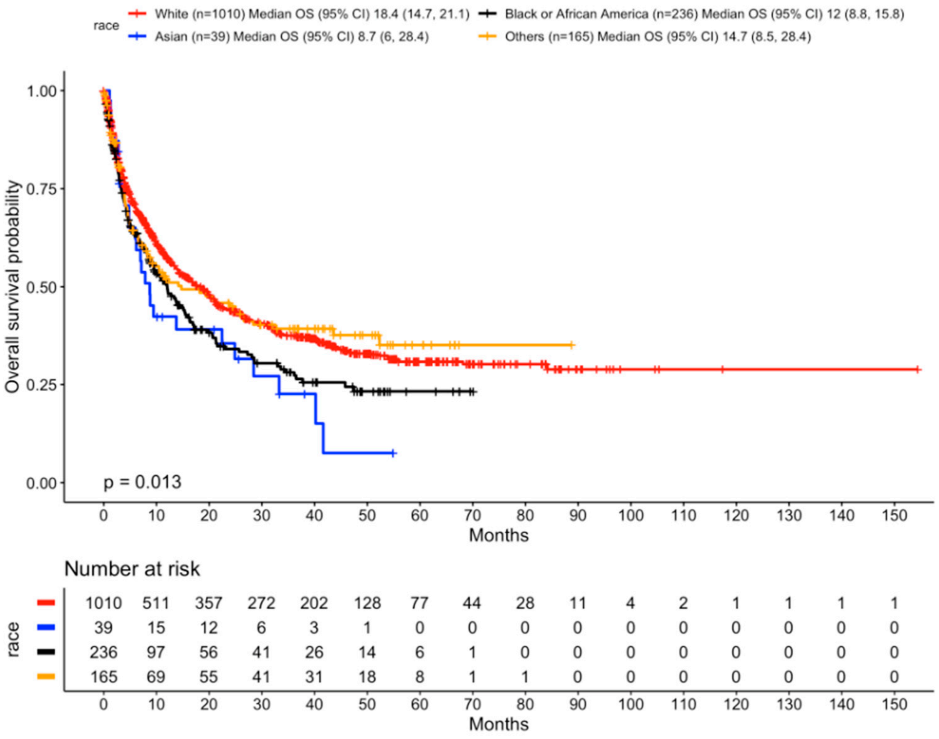

C. Gender

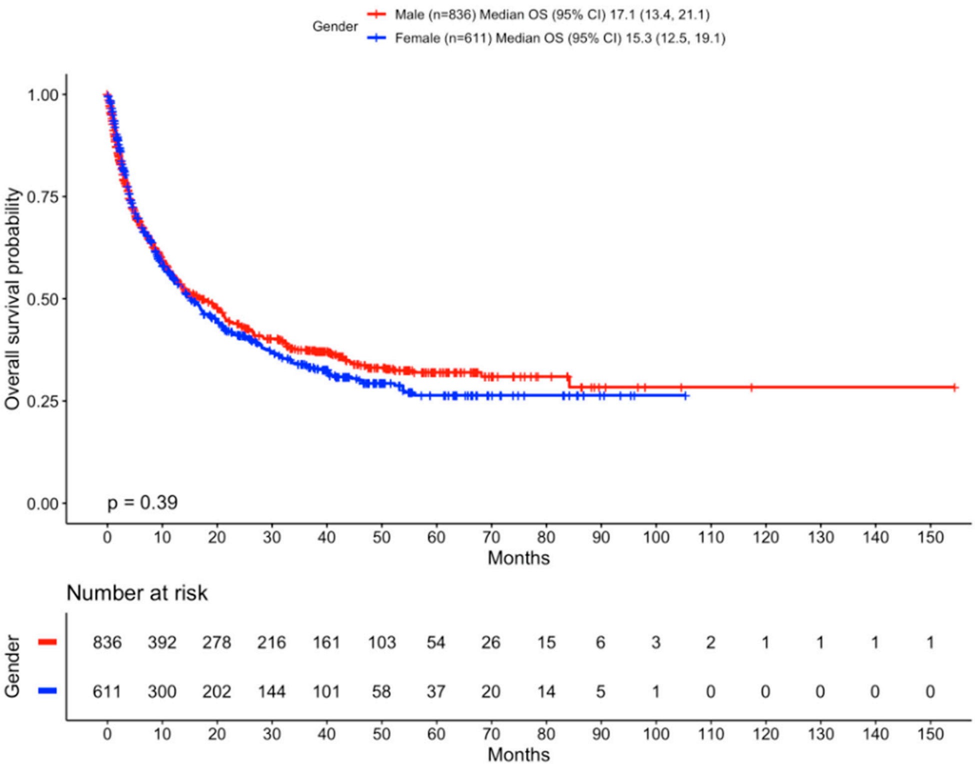

D. History of AID

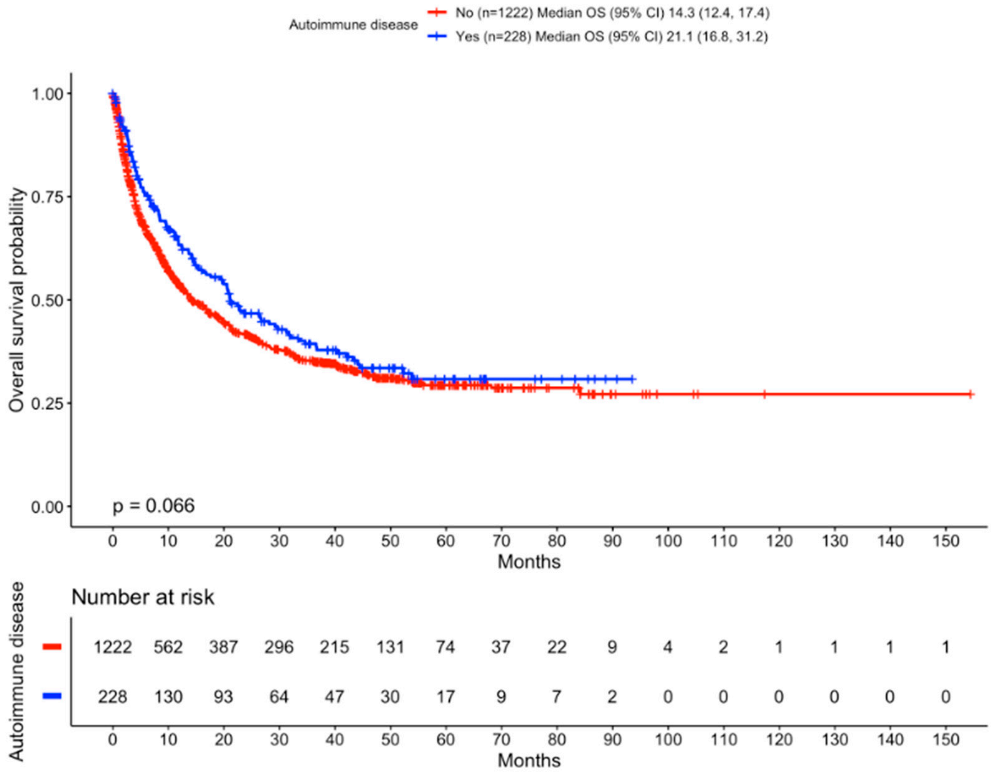

E. BMI <30 kg/m^2 vs. BMI ≥30 kg/m^2

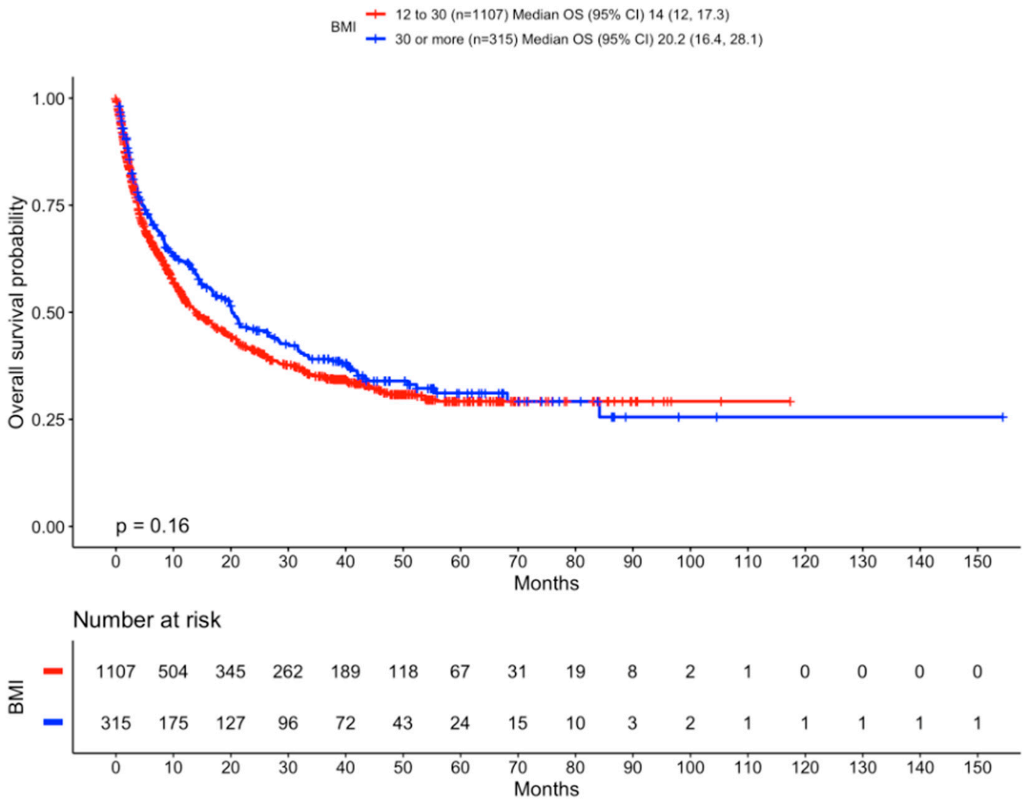

F. Smoking

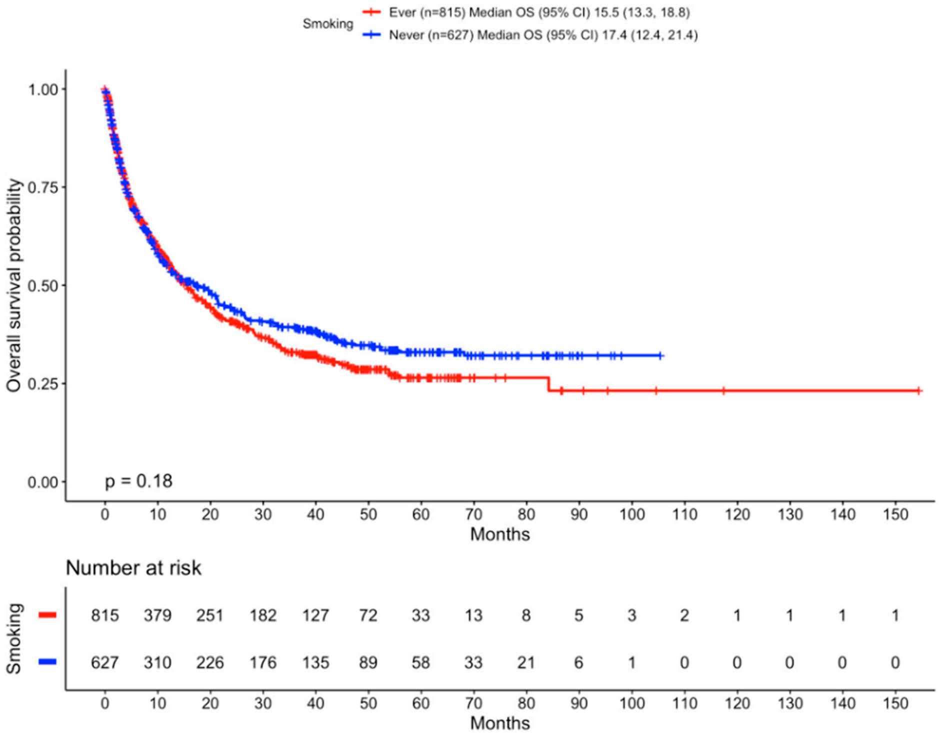

G. Combined CVI

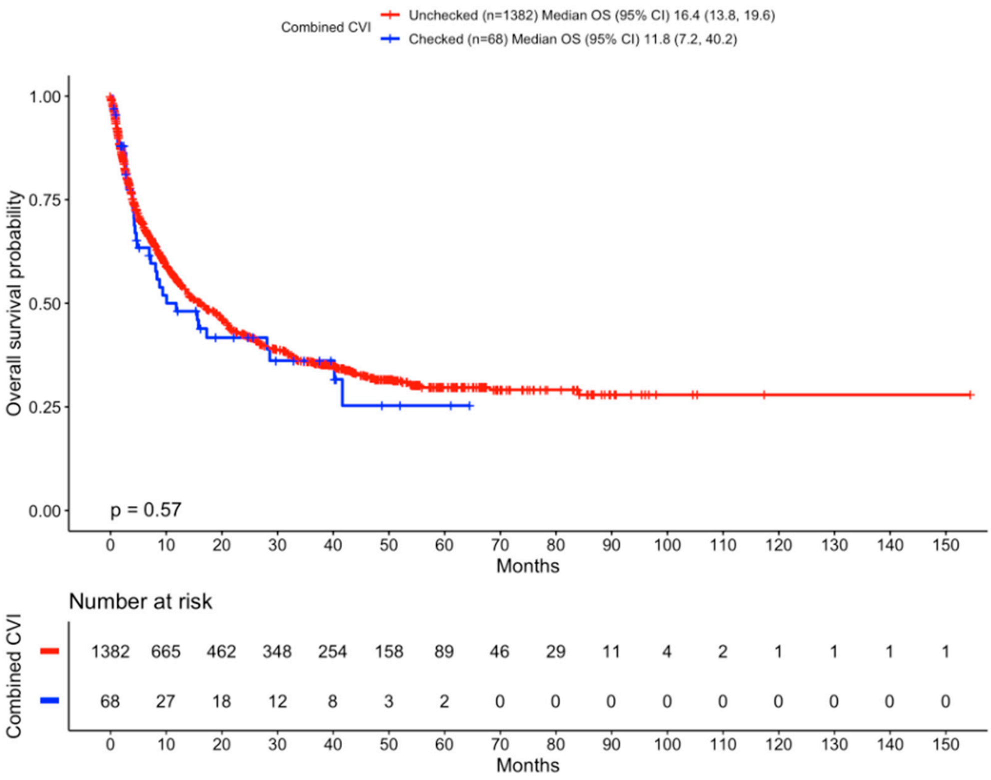

H. Number of metastatic sites

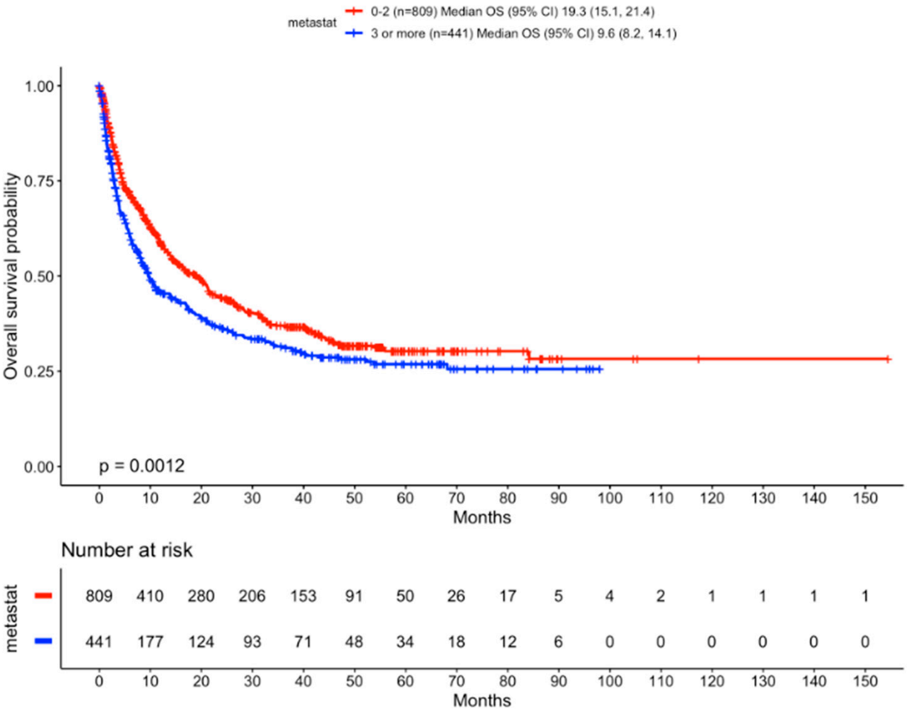

Figure S4. Overall survival probability in NSCLC patients treated with ICIs by unique cohorts.

A. Age ≤75 years vs. Age >75 years

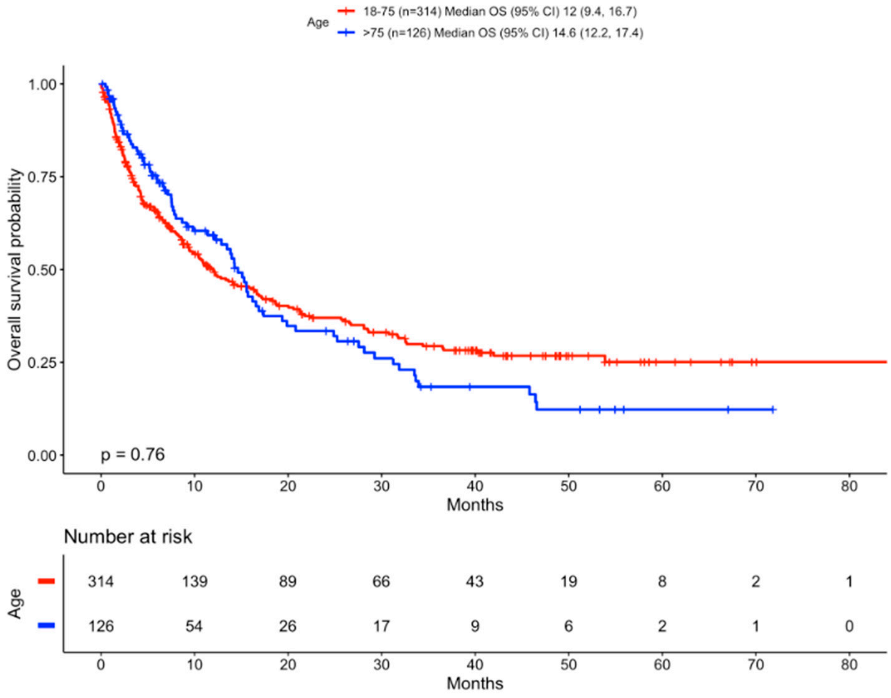

B. Race

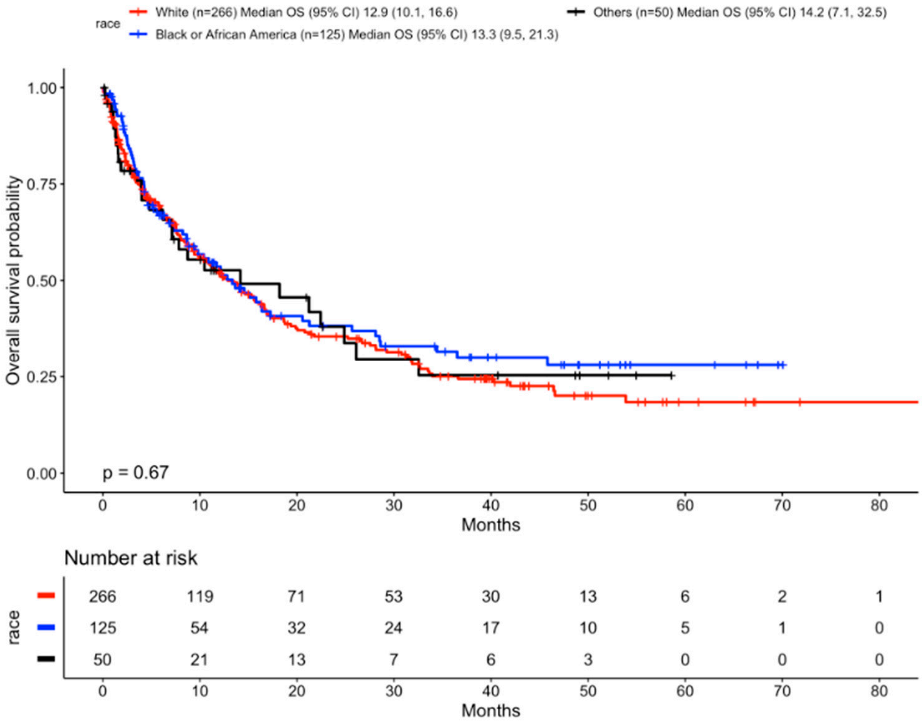

C. Gender

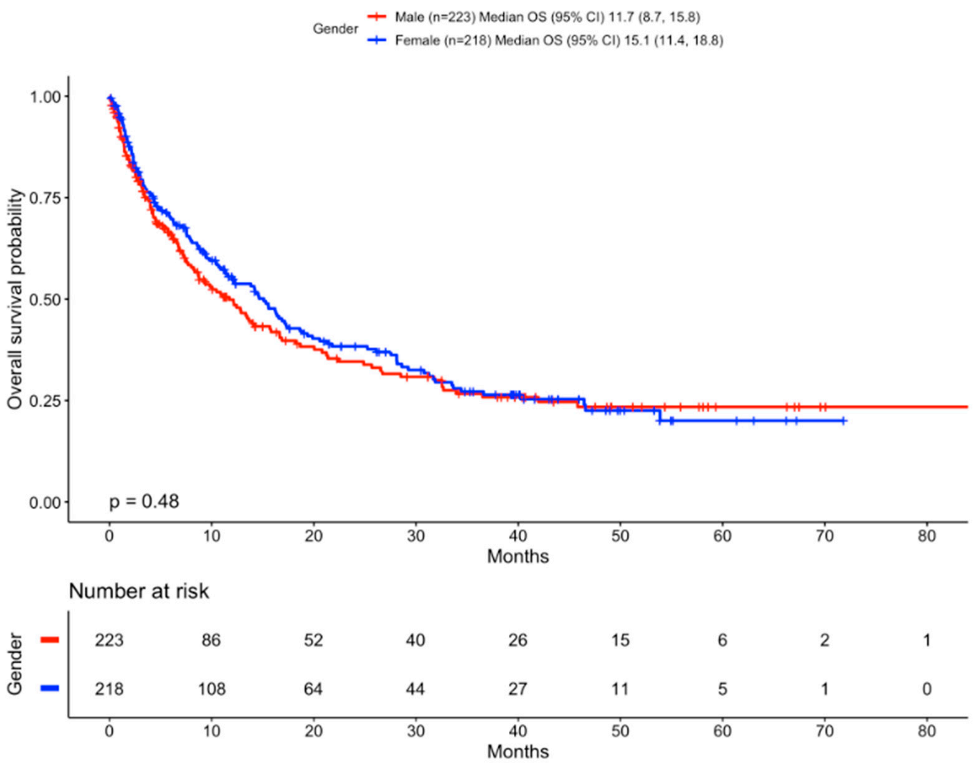

D. History of AID

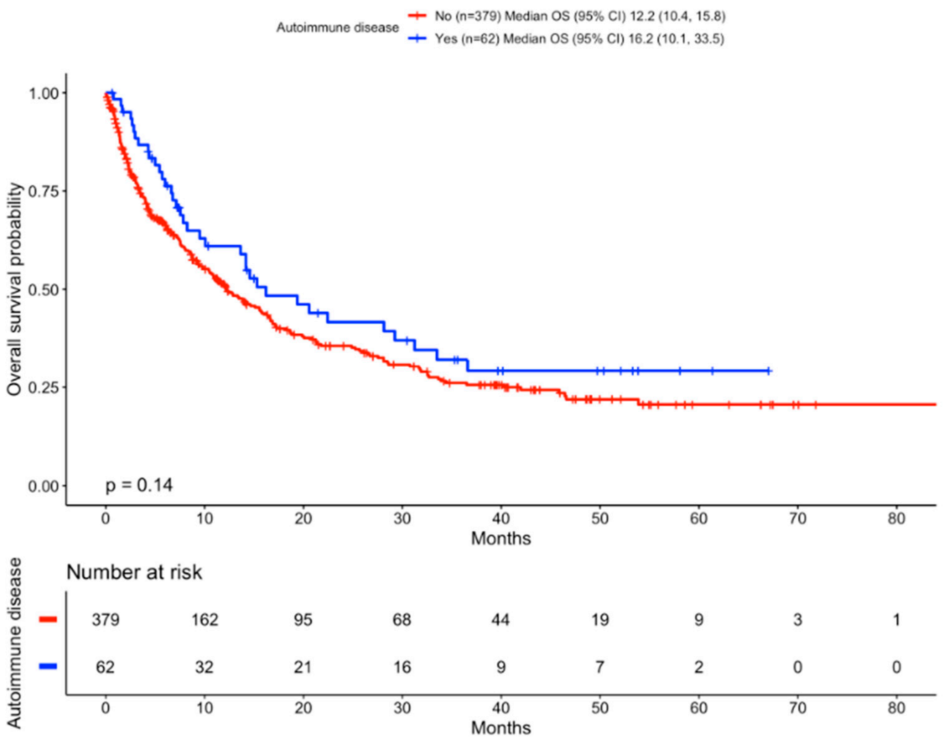

E. BMI <30 kg/m^2 vs. BMI ≥30 kg/m^2

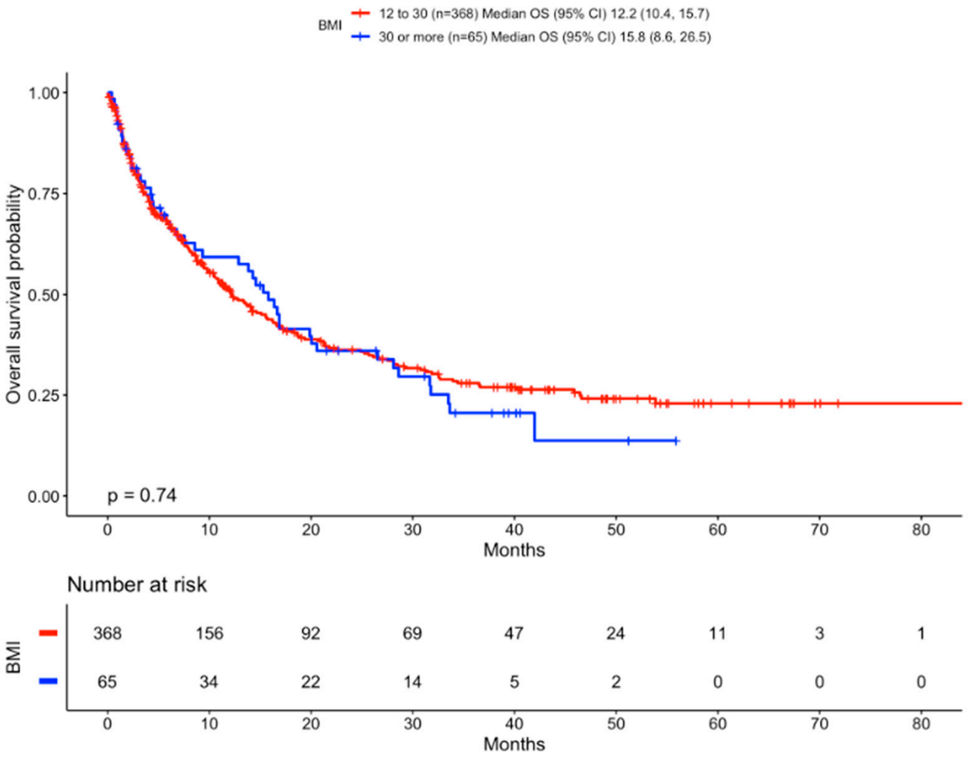

F. Smoking

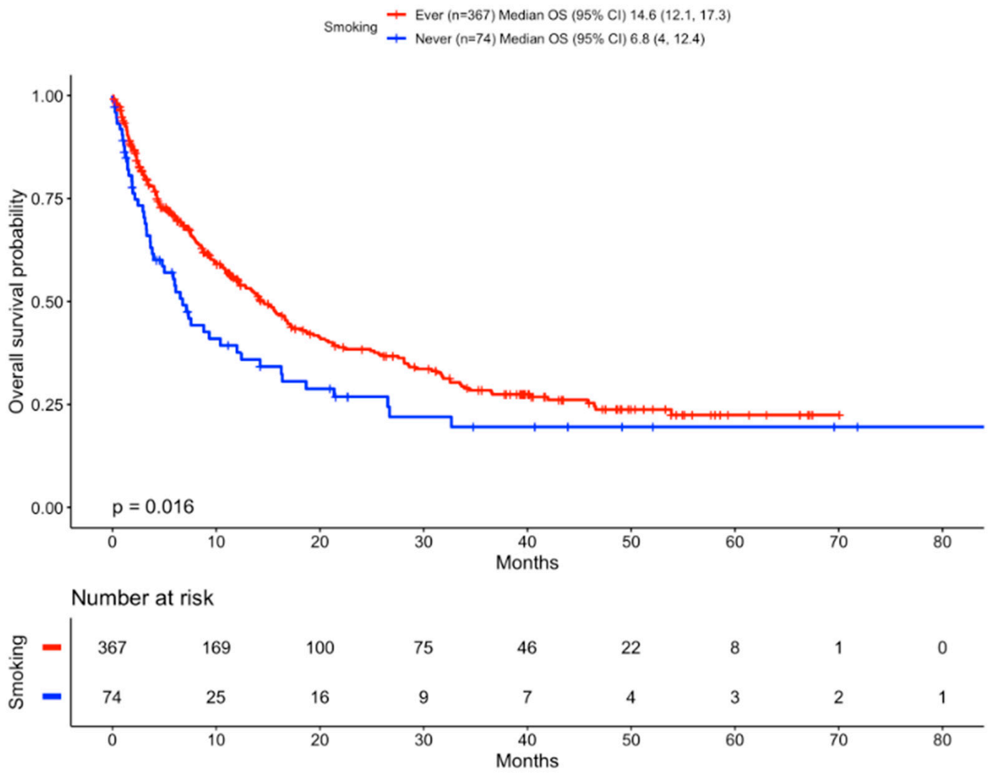

G. Combined CVI

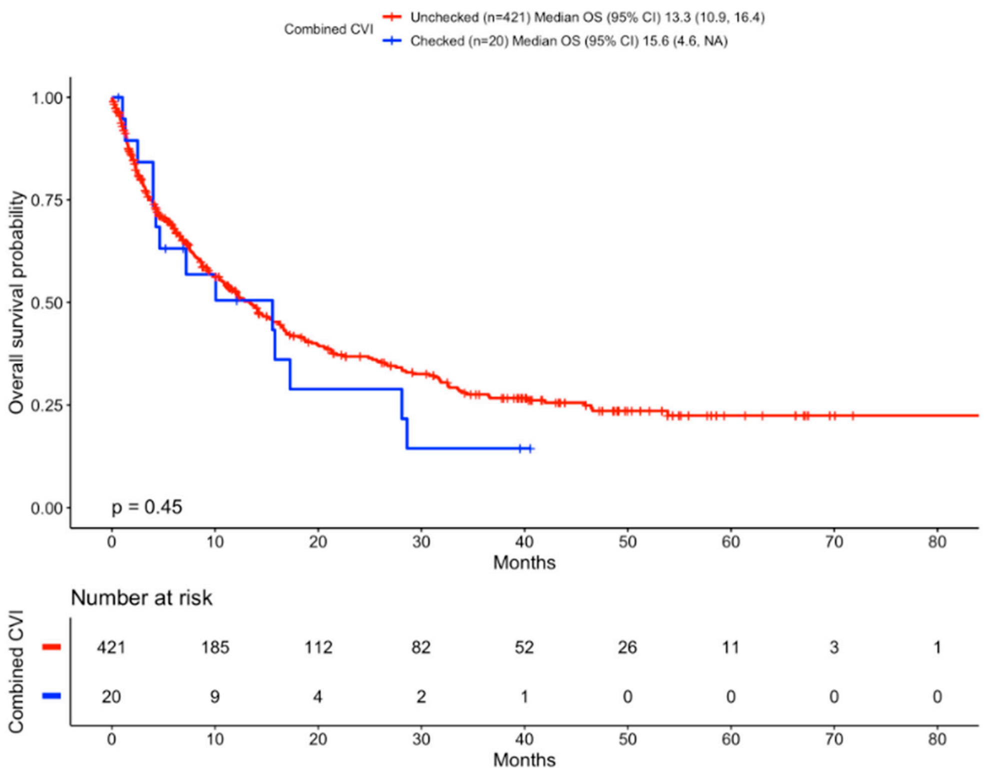

H. Number of metastatic sites

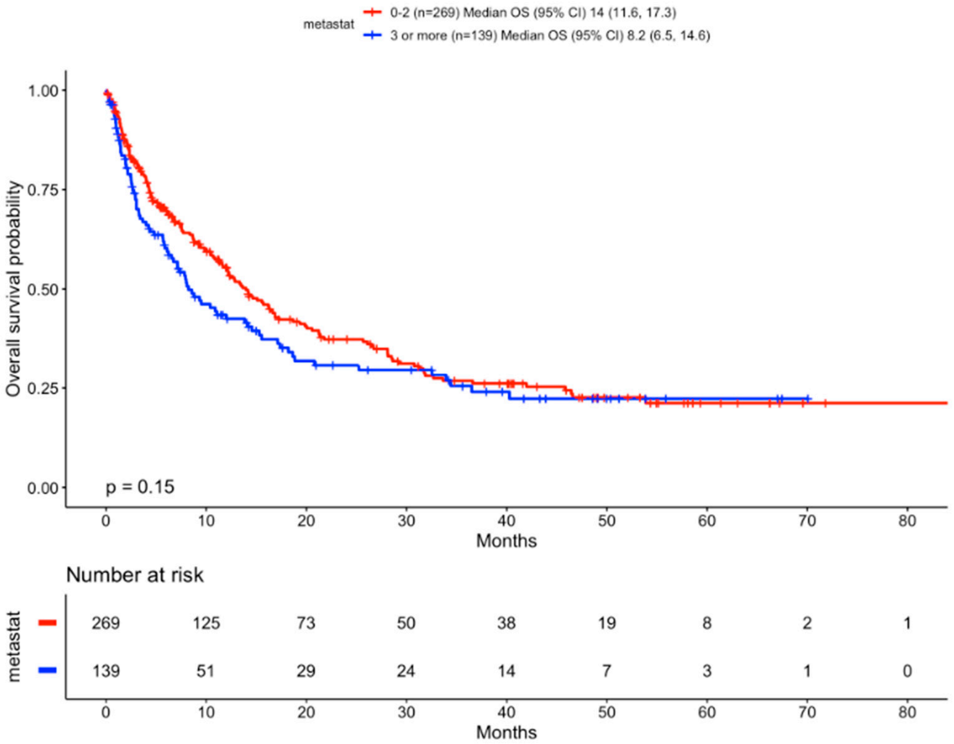

Figure S5. Overall survival probability in NSCLC patients treated with PD-(L)1 monotherapy by unique cohorts.

A. Age ≤75 years vs. Age >75 years

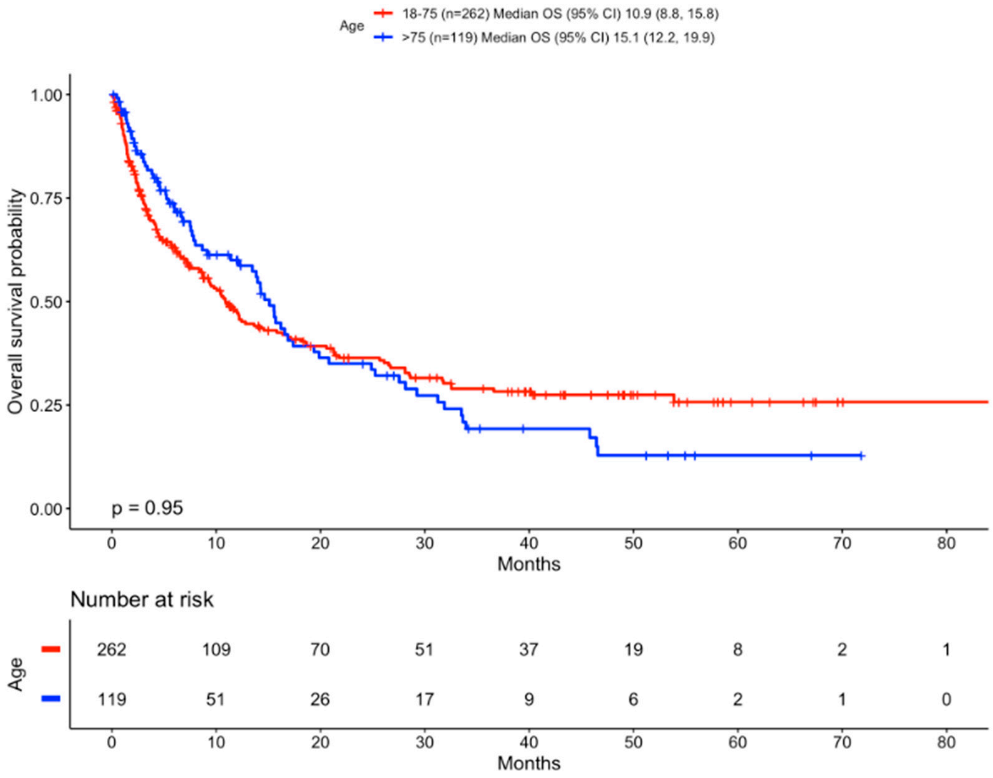

B. Race

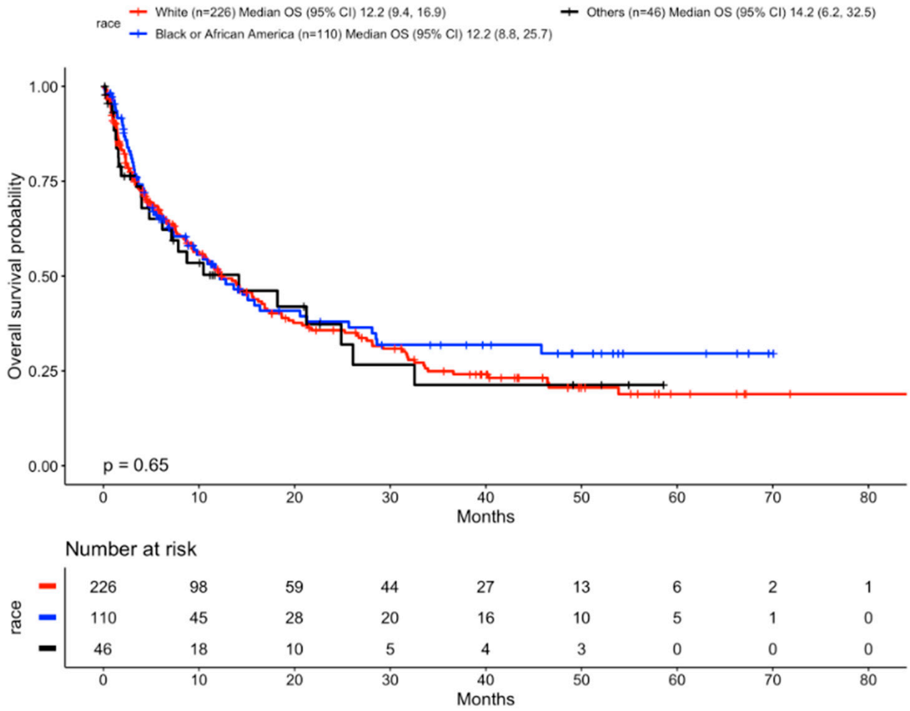

C. Gender

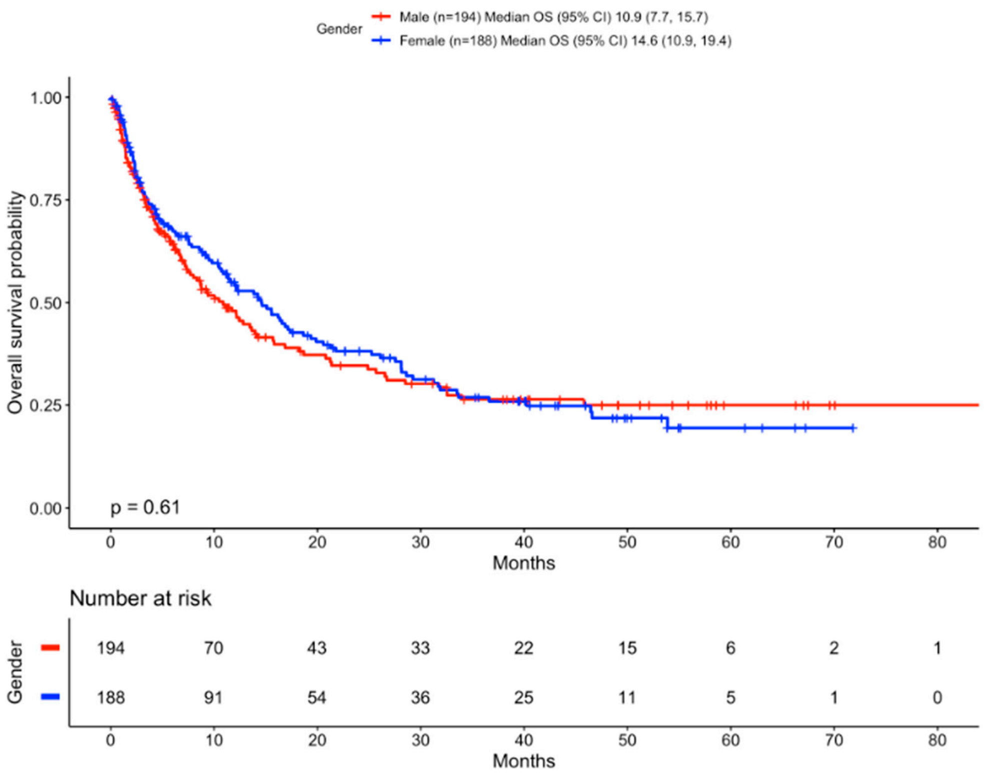

D. History of AID

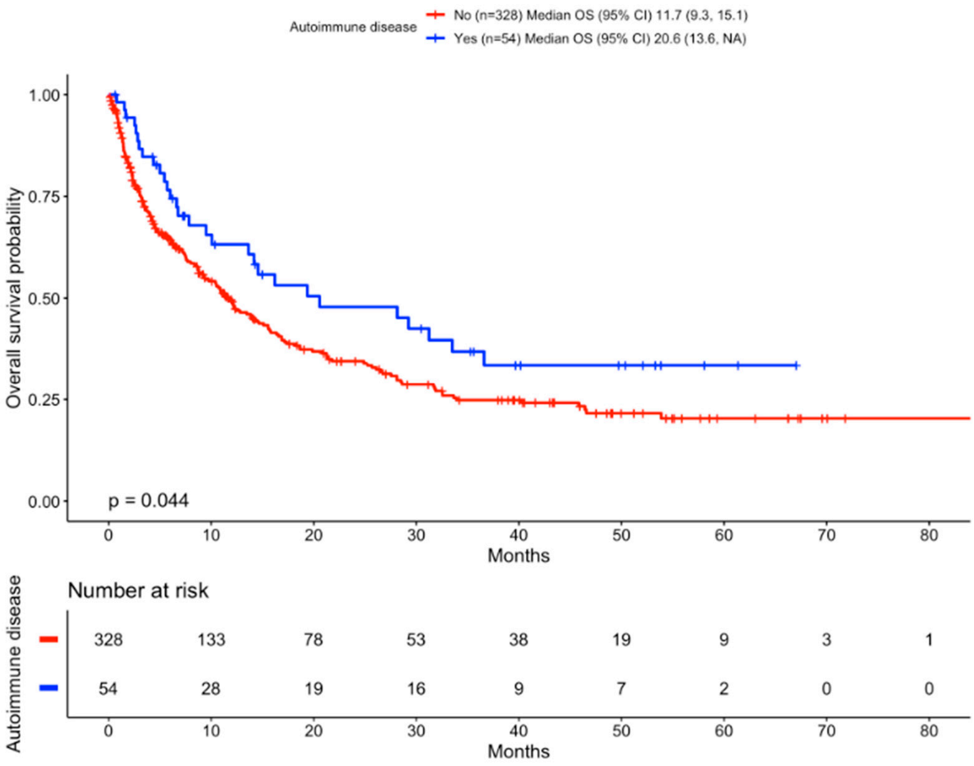

E. BMI <30 kg/m^2 vs. BMI ≥30 kg/m^2

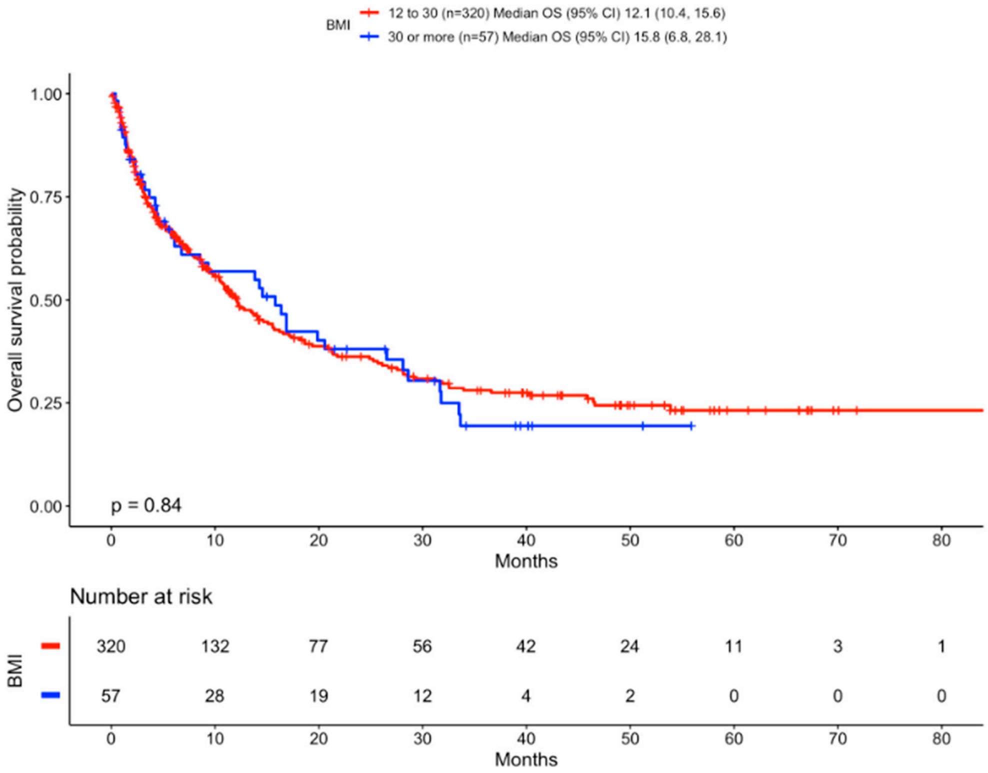

F. Smoking

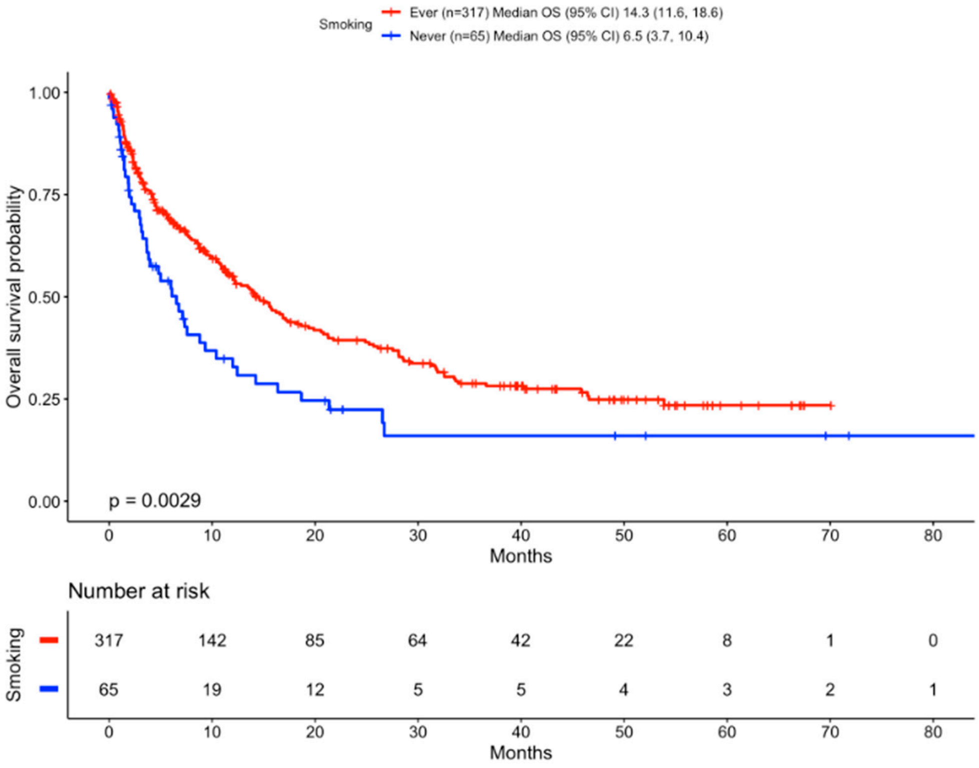

G. Combined CVI

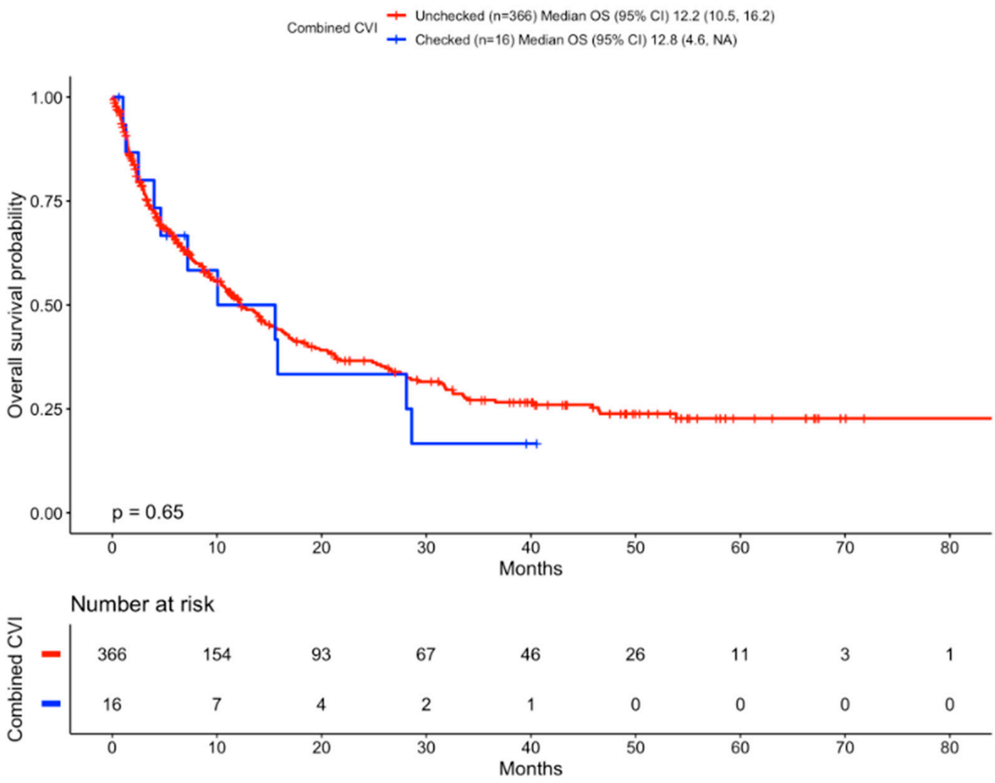

H. Number of metastatic sites

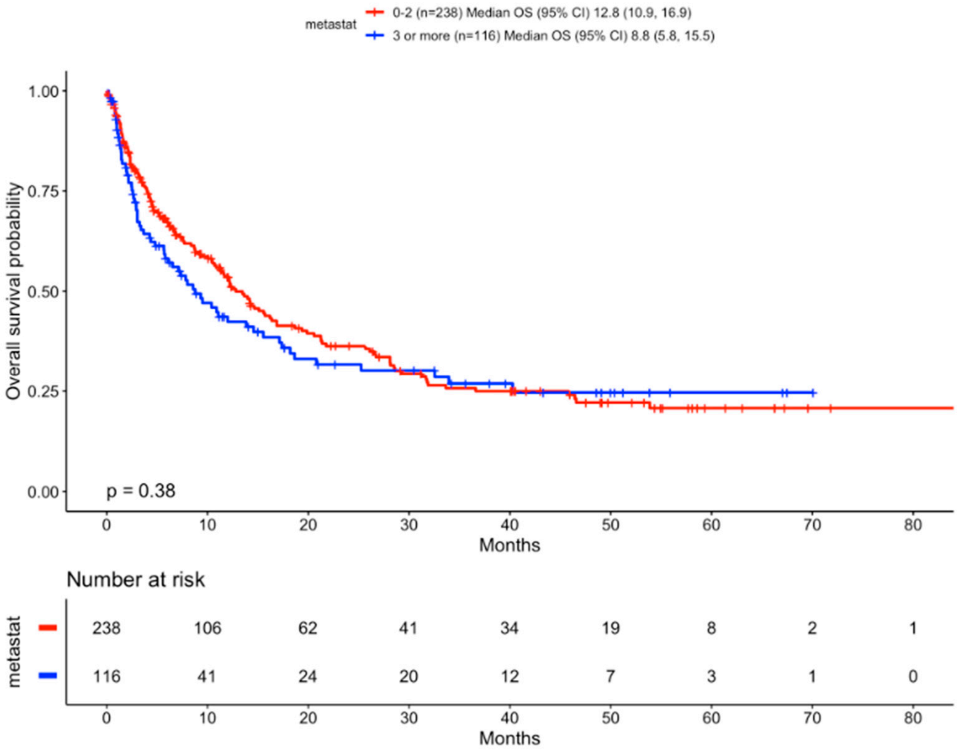

Figure S6. Overall survival hazard ratio by unique patient groups of interest in White patients in the anti-PD-(L)1 monotherapy NSCLC cohort.

Variable		N	Hazard ratio		p
**Age, years**	18-75	131		Reference	
	>75	71		1.16 (0.80, 1.69)	0.439
**Gender**	Male	92		Reference	
	Female	110		0.73 (0.51, 1.05)	0.088
**Autoimmune disease**	No	169		Reference	
	Yes	33		0.60 (0.35, 1.01)	0.056
**Pre-treatment ECOG PS**	0 - 1	145		Reference	
	2 or more	57		1.74 (1.19, 2.54)	0.004
**Lines of therapy**	1	48		Reference	
	2	108		1.44 (0.91, 2.27)	0.116
	>=3	46		1.91 (1.13, 3.24)	0.015

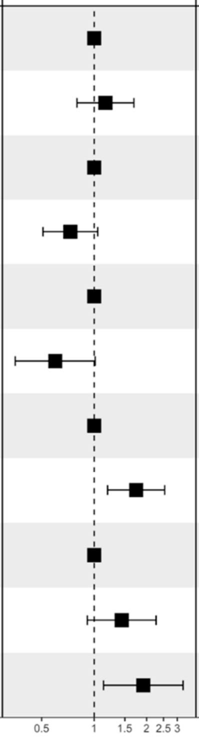

May and Hosmer goodness-of-fit test *p*=0.457.

Figure S7. Overall survival hazard ratio by unique patient groups of interest in Black patients in the anti-PD-(L)1 monotherapy NSCLC cohort.

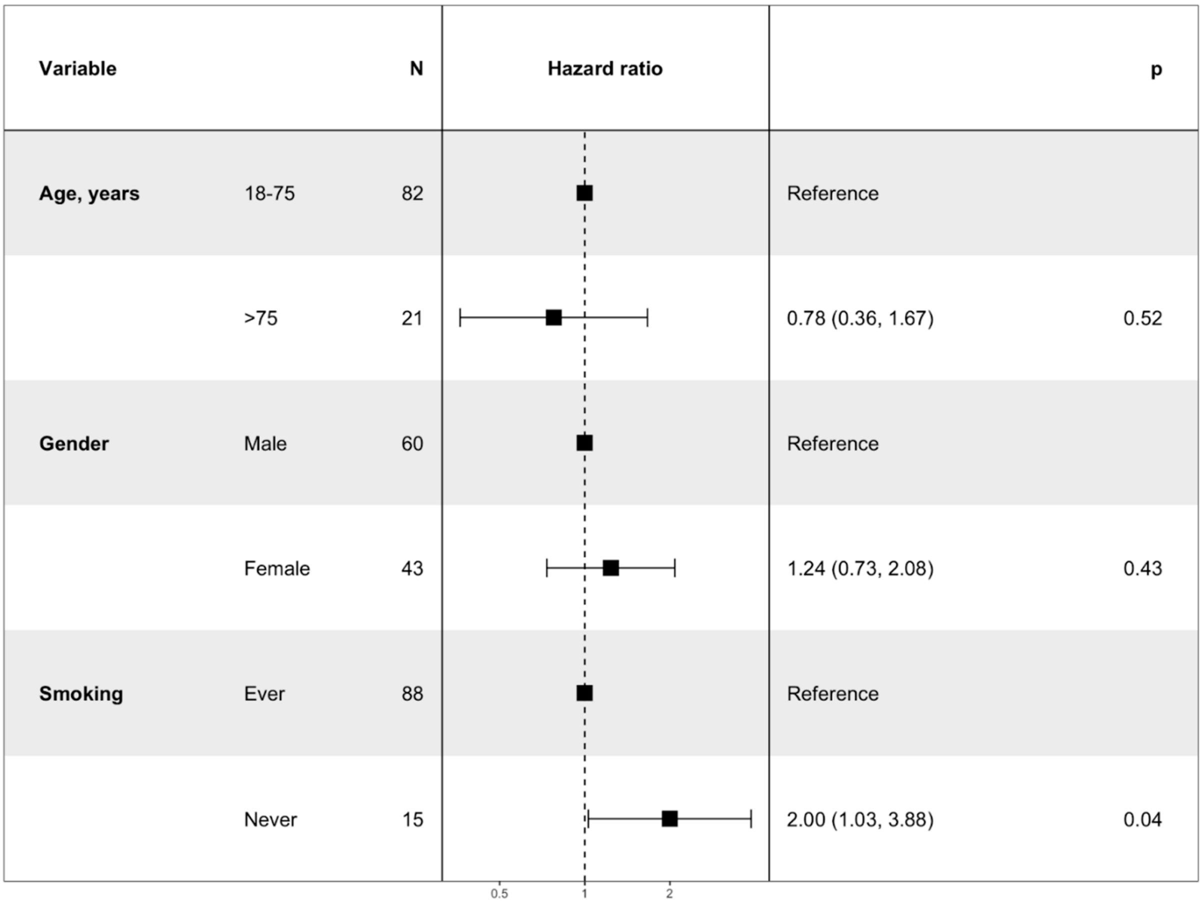

May and Hosmer goodness-of-fit test p = 0.77.

Figure S8. Time to treatment failure (TTF) probability by ECOG performance status and lines of therapy in the entire cohort, entire NSCLC cohort, and PD-(L)1 monotherapy cohort.

A. ECOG PS≤1 vs. ECOG PS≥2 in entire cohort

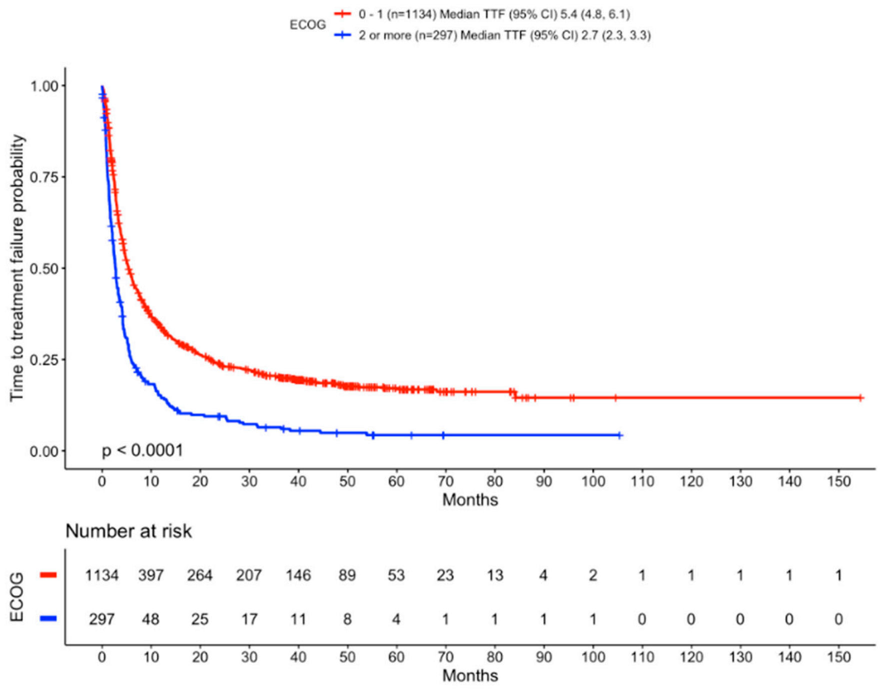

B. ECOG PS≤1 vs. ECOG PS≥2 in entire NSCLC cohort

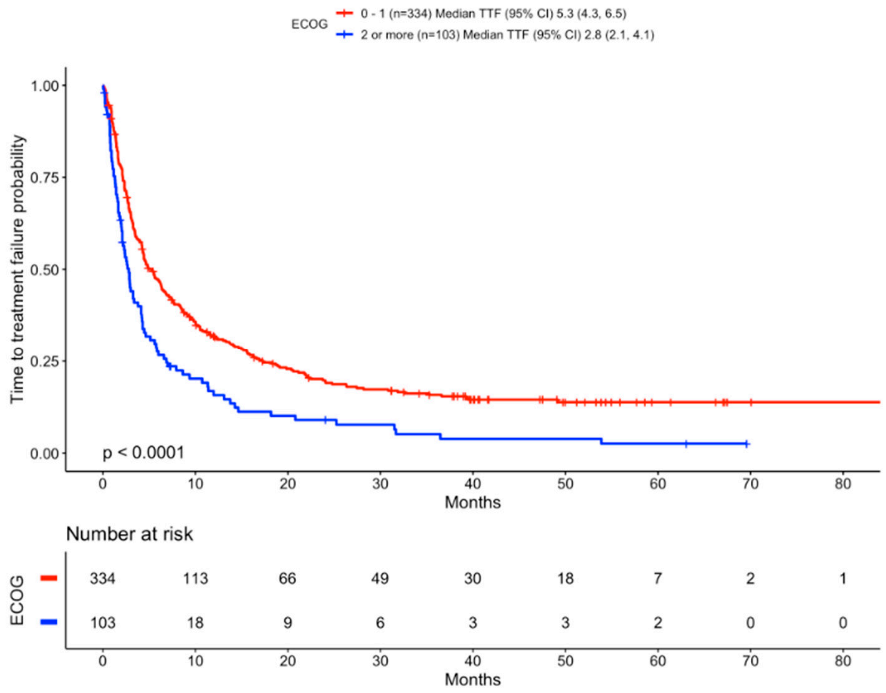

C. ECOG PS≤1 vs. ECOG PS≥2 in NSCLC PD-L1 monotherapy cohort

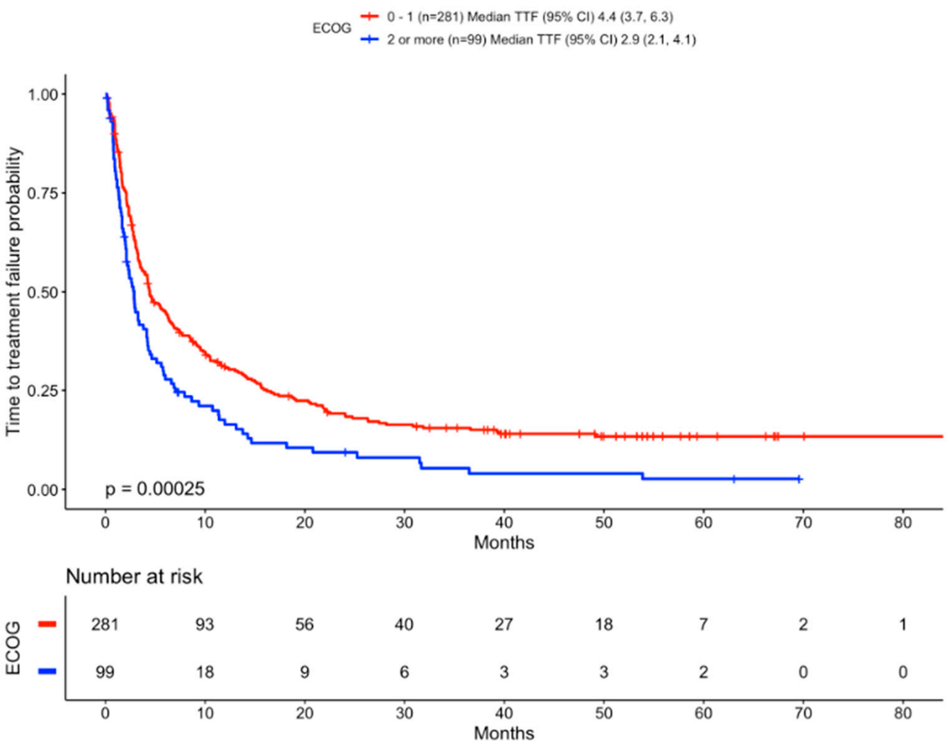

D. Lines of therapy in entire cohort

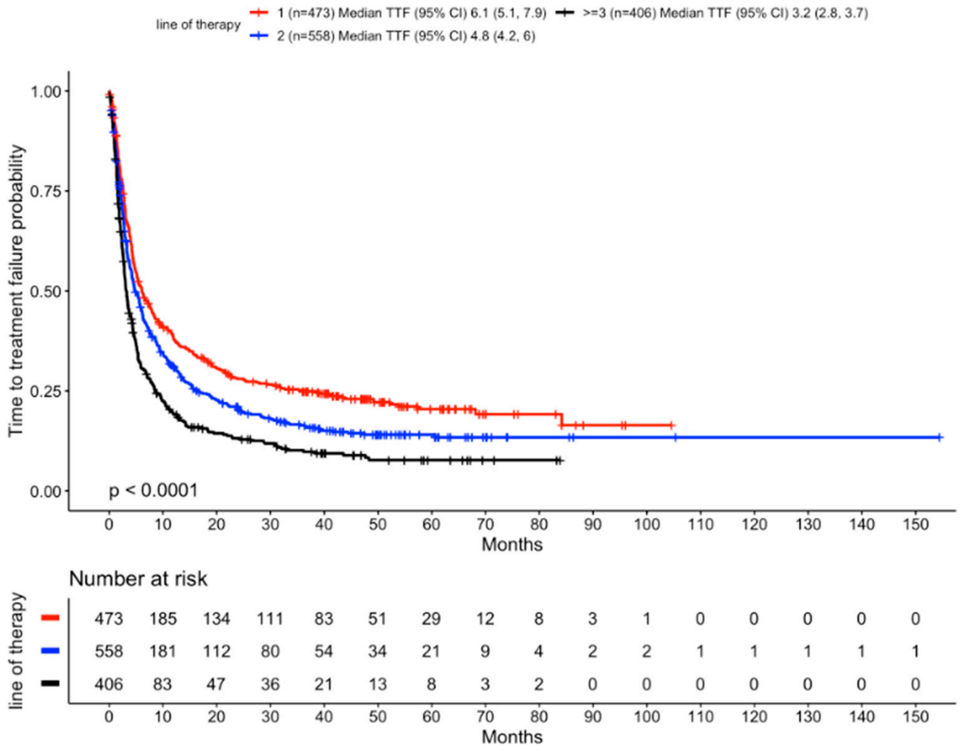

E. Lines of therapy in entire NSCLC cohort

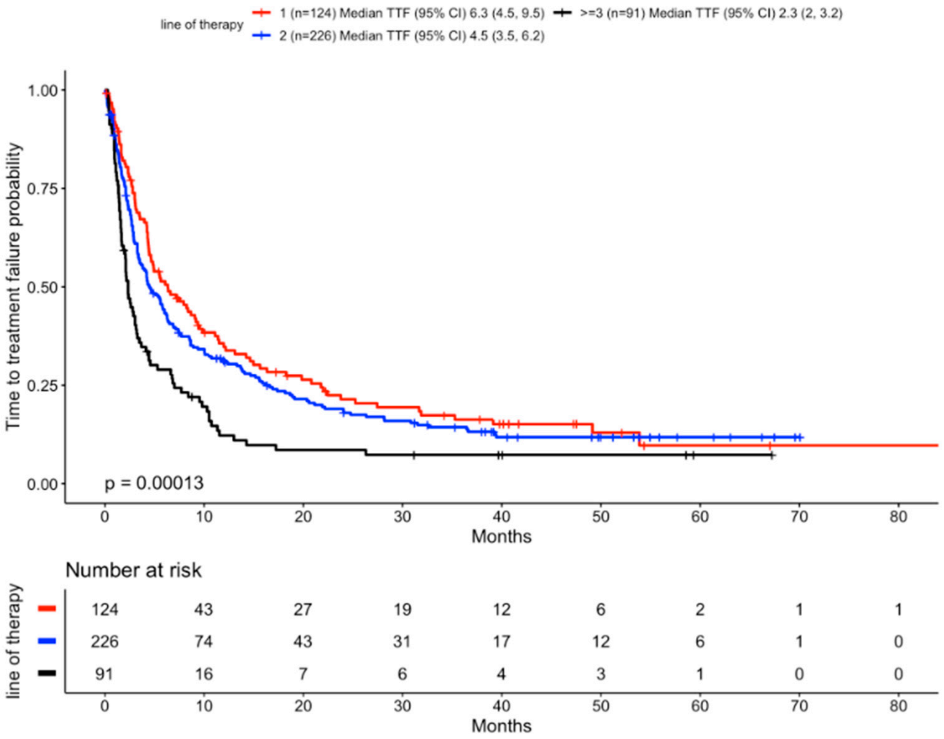

F. Lines of therapy in NSCLC PD-L1 monotherapy cohort

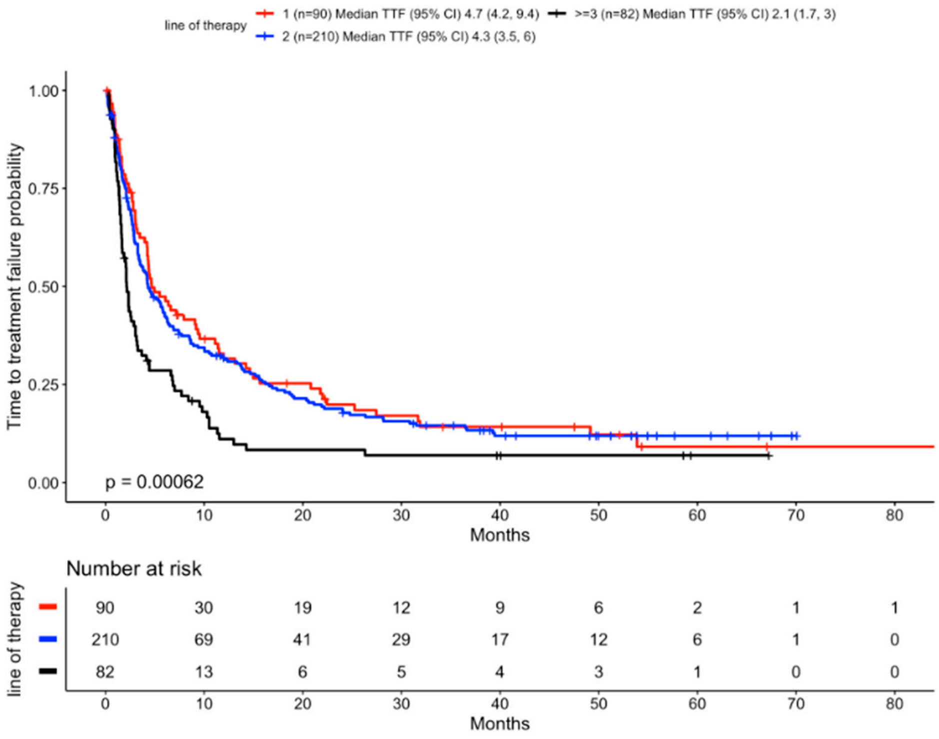

Figure S9. Time to treatment failure (TTF) probability in patients treated with ICIs by unique cohorts.

A. Age ≤75 years vs. Age >75 years

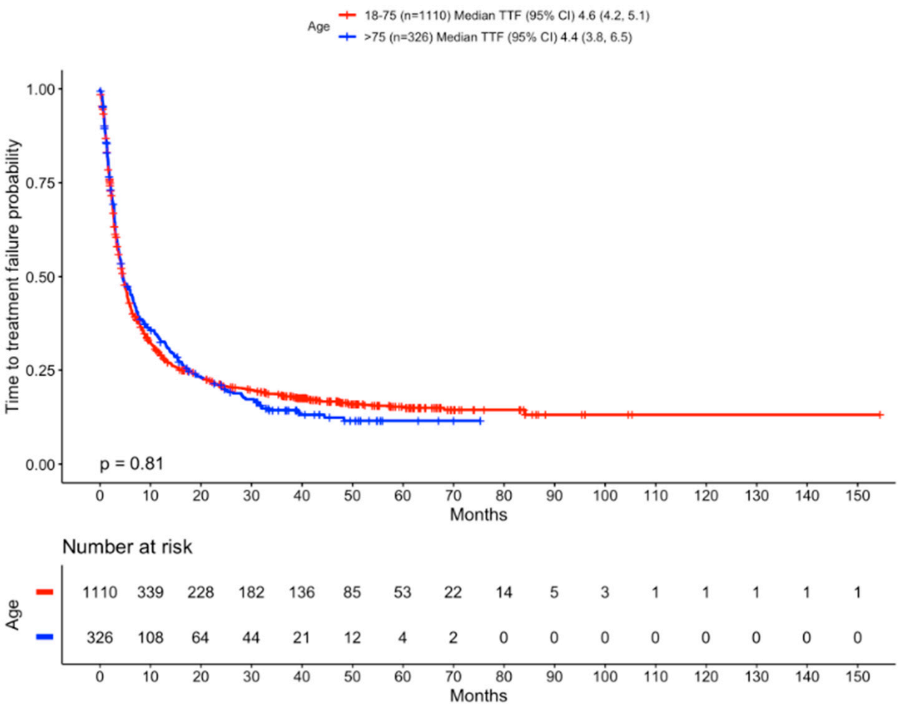

B. Race

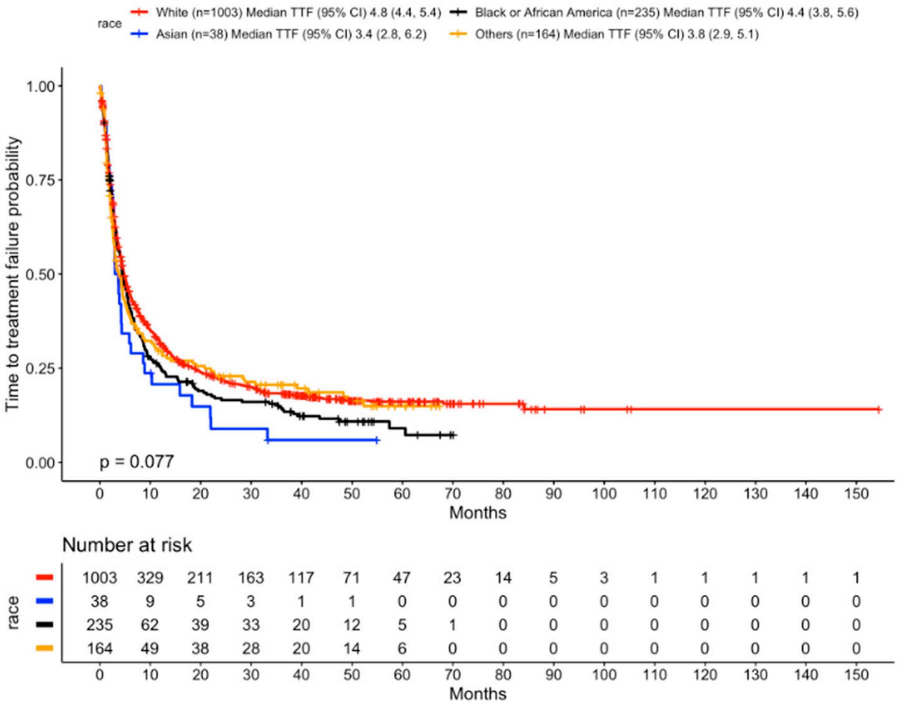

C. Gender

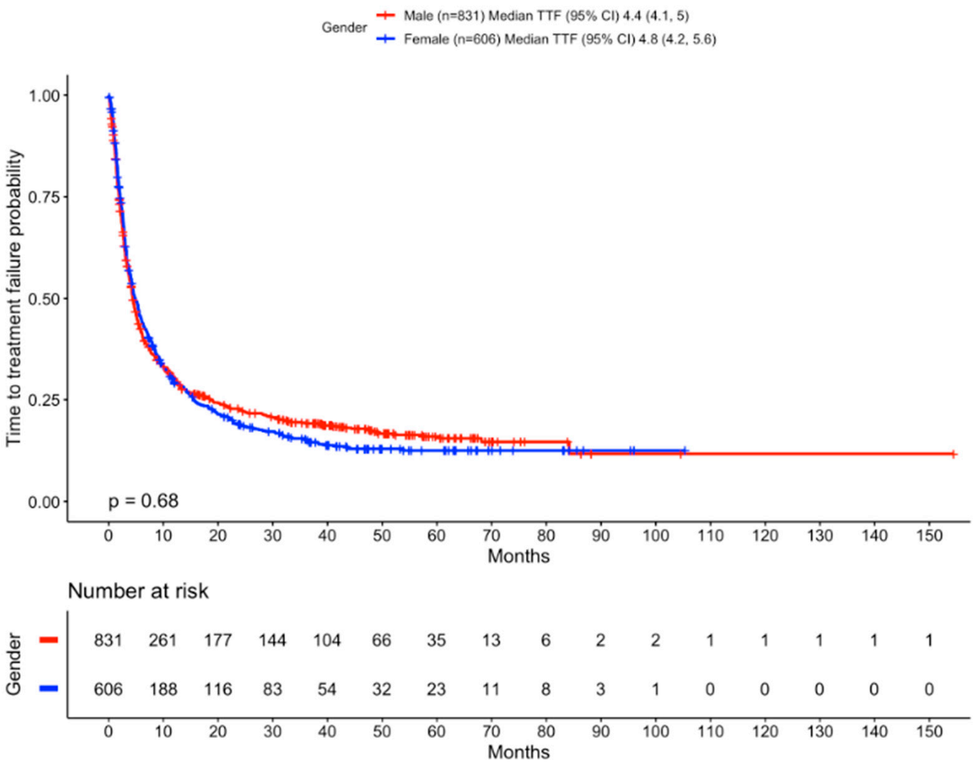

D. History of AID

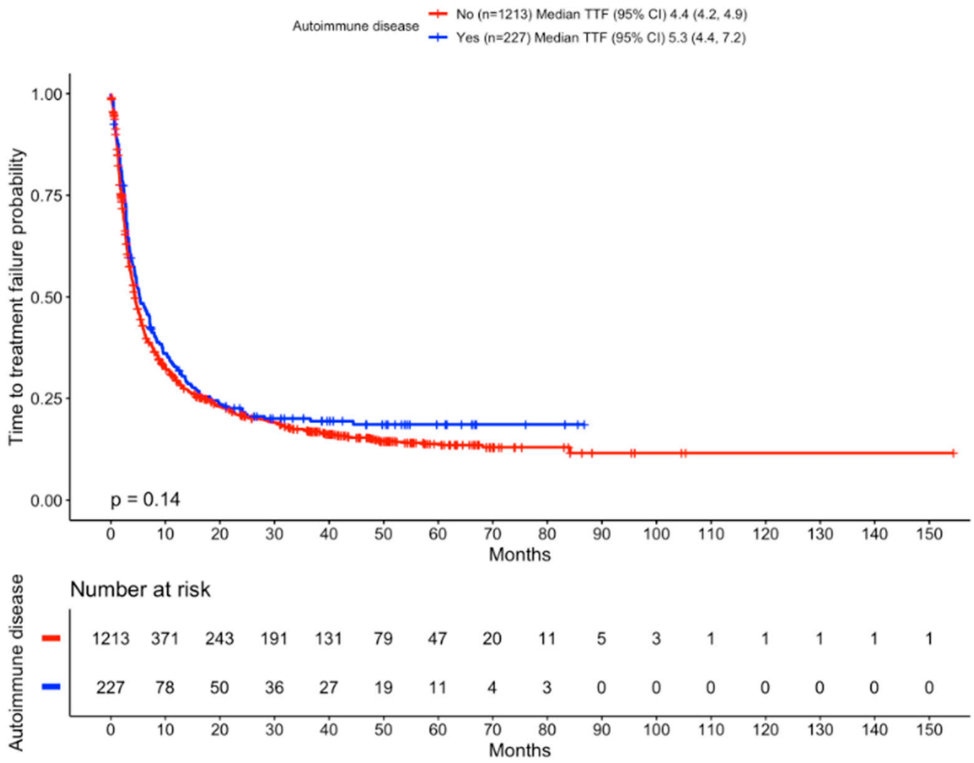

E. BMI<30 kg/m^2 vs. BMI ≥30 kg/m^2

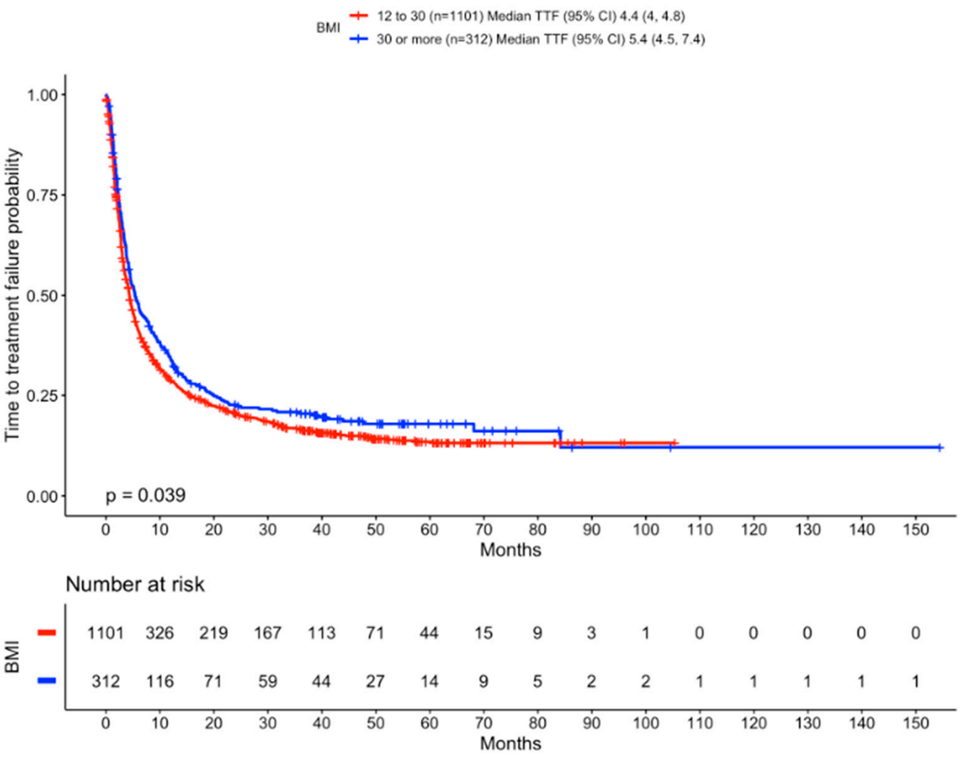

F. Smoking

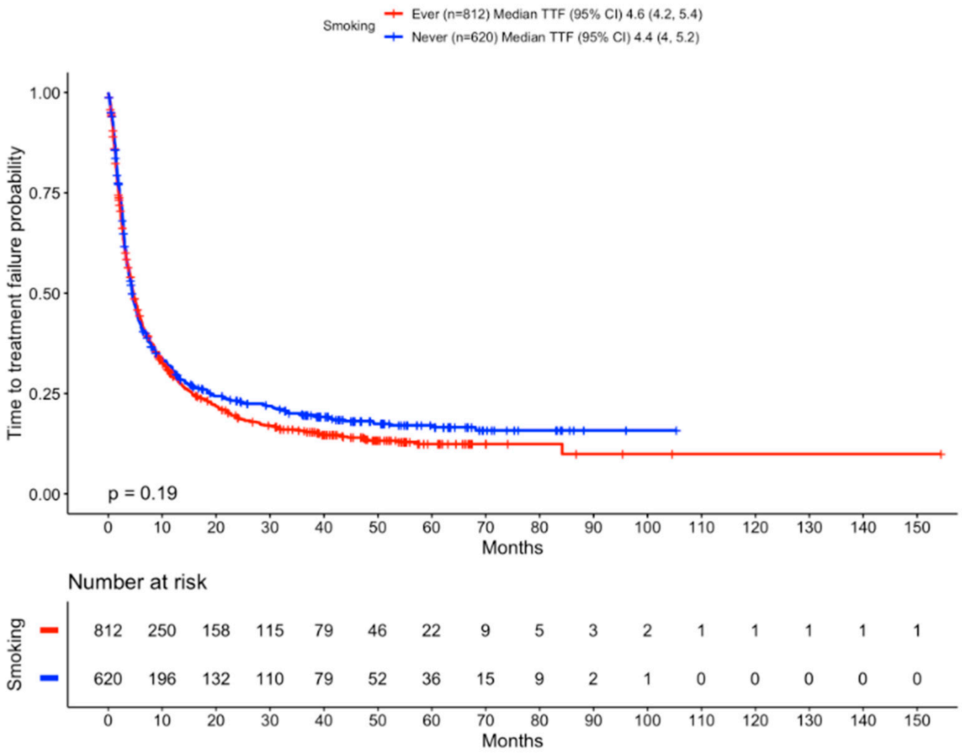

G. Combined CVI

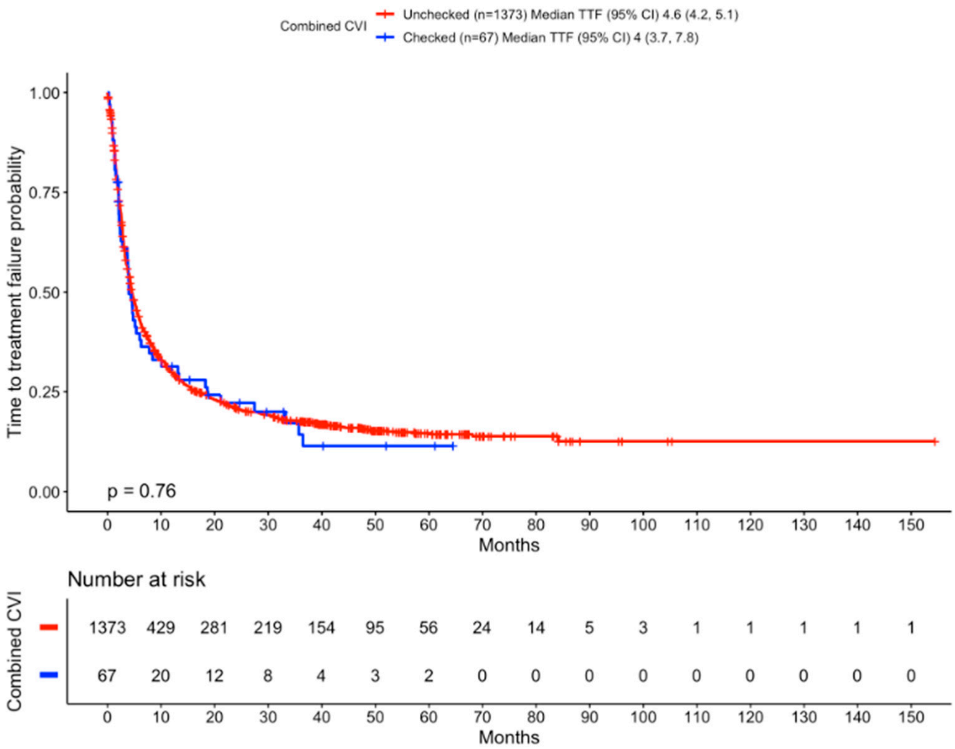

H. Number of metastatic sites

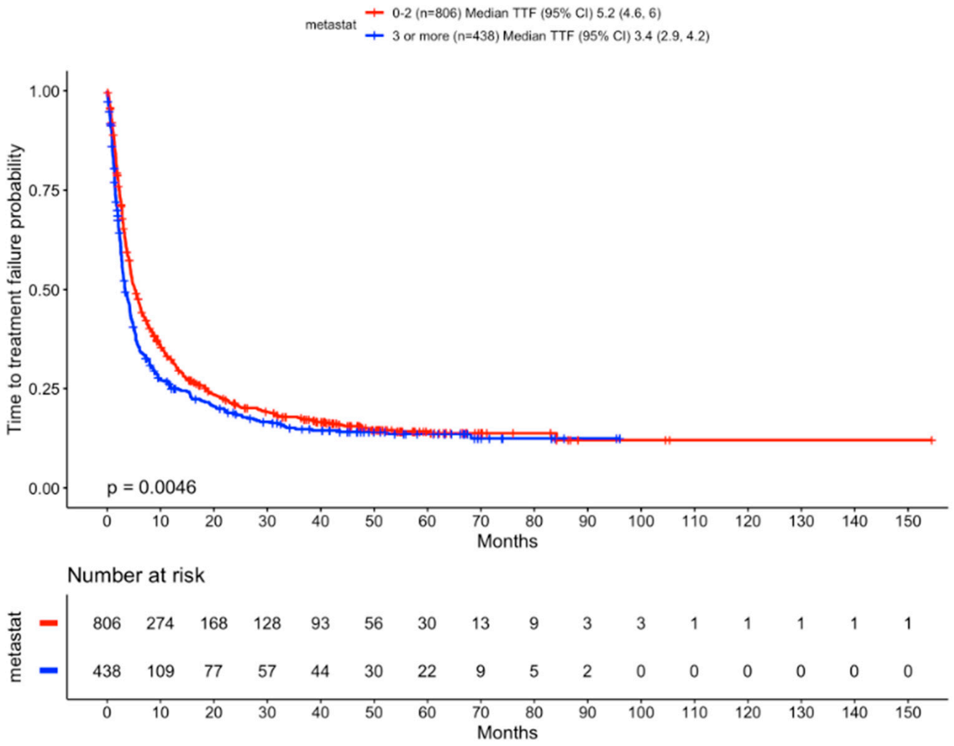

Figure S10. Time to treatment failure (TTF) probability in NSCLC patients treated with ICIs by unique cohorts.

A. **Age ≤75 years vs. Age >75 years**

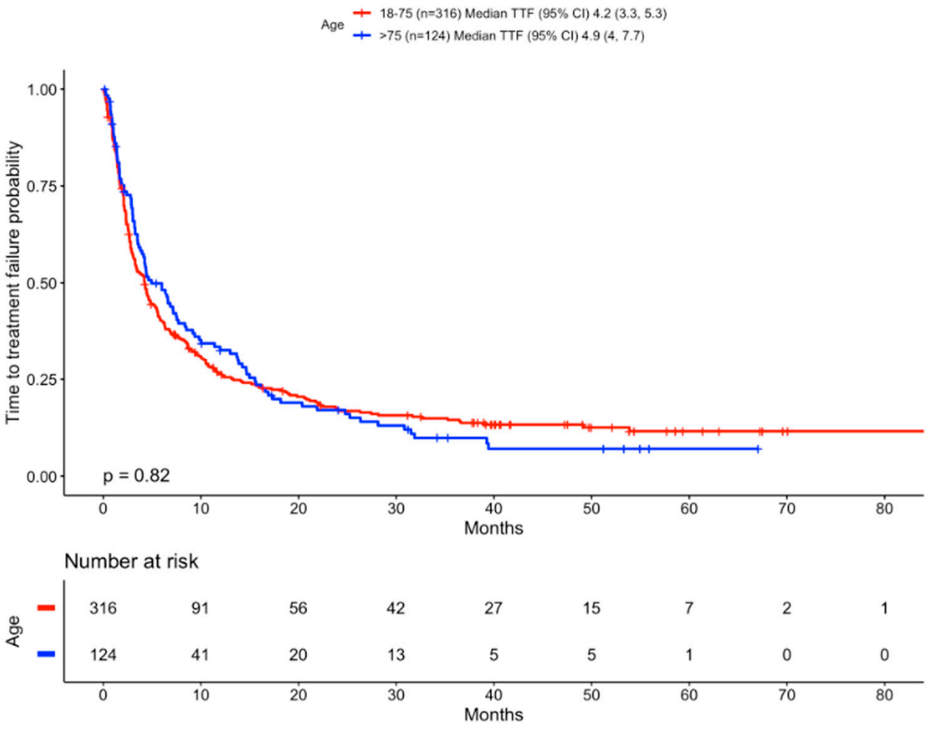

B. **Race**

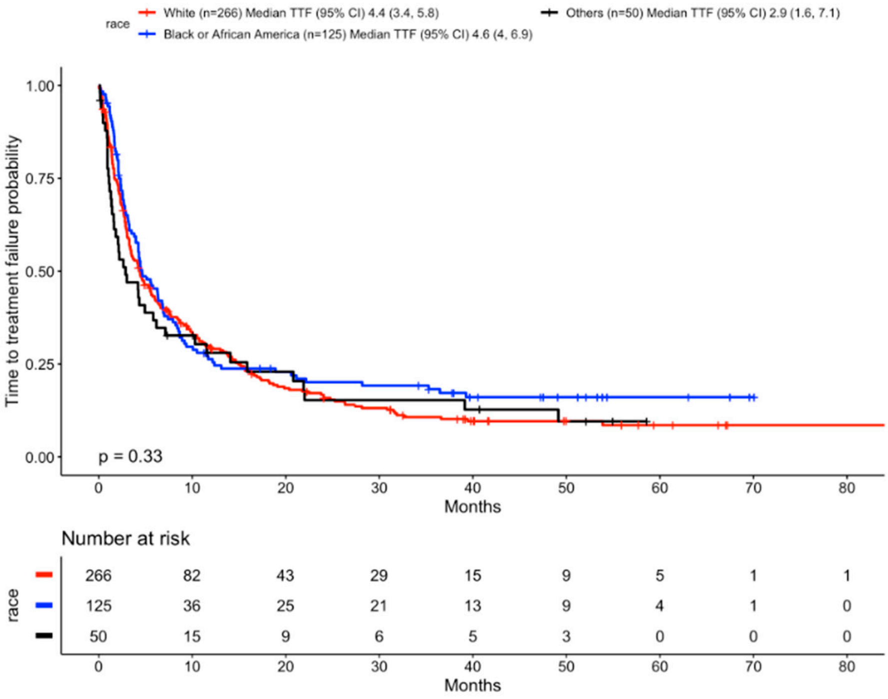

C. Gender

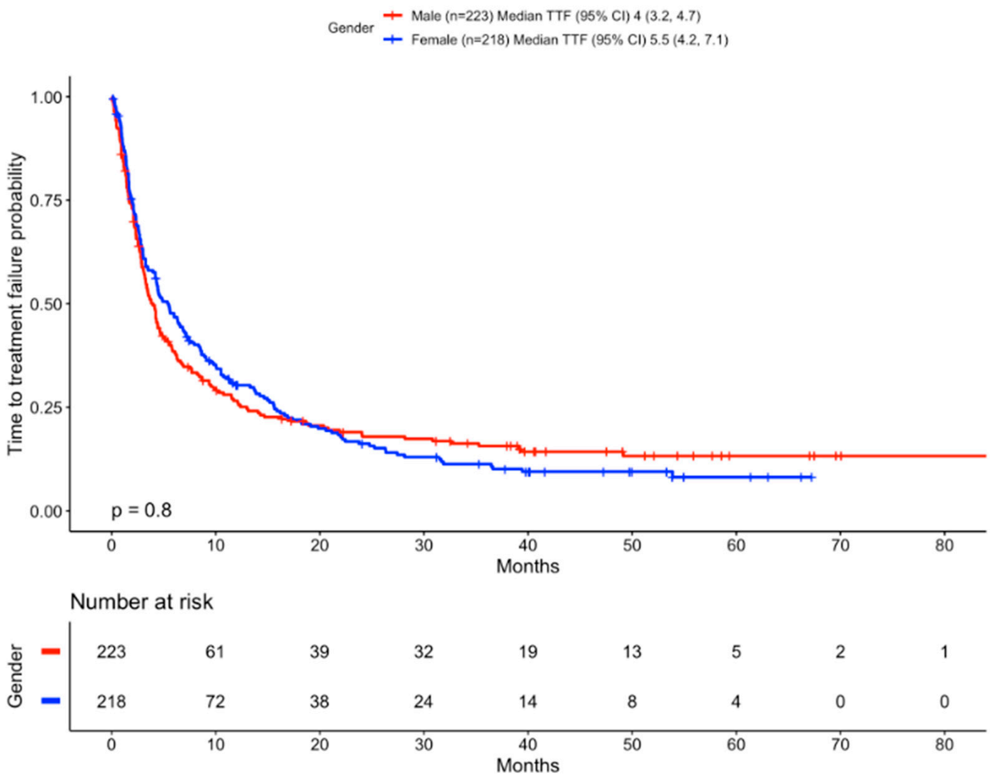

D. History of AID

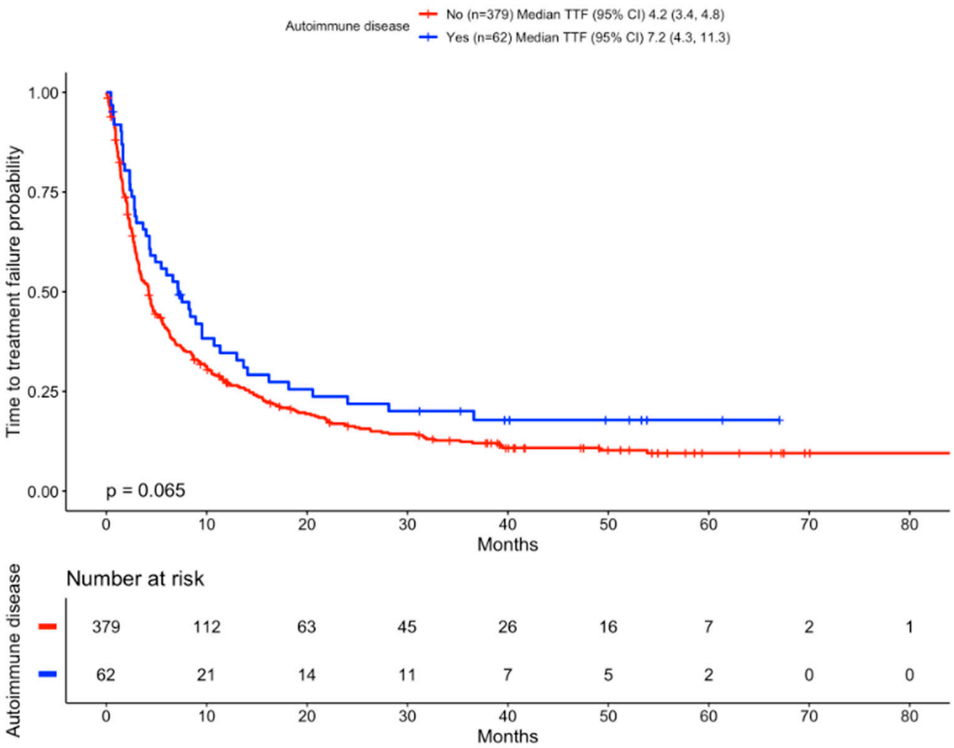

E. BMI<30 kg/m² vs. BMI ≥30 kg/m²

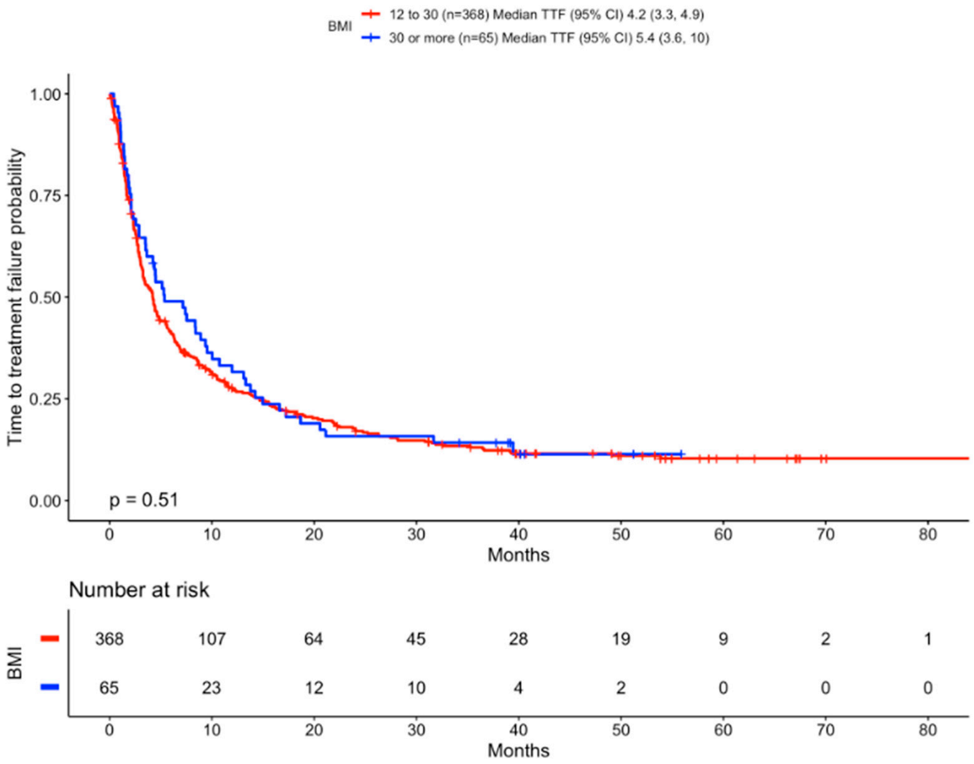

F. Smoking

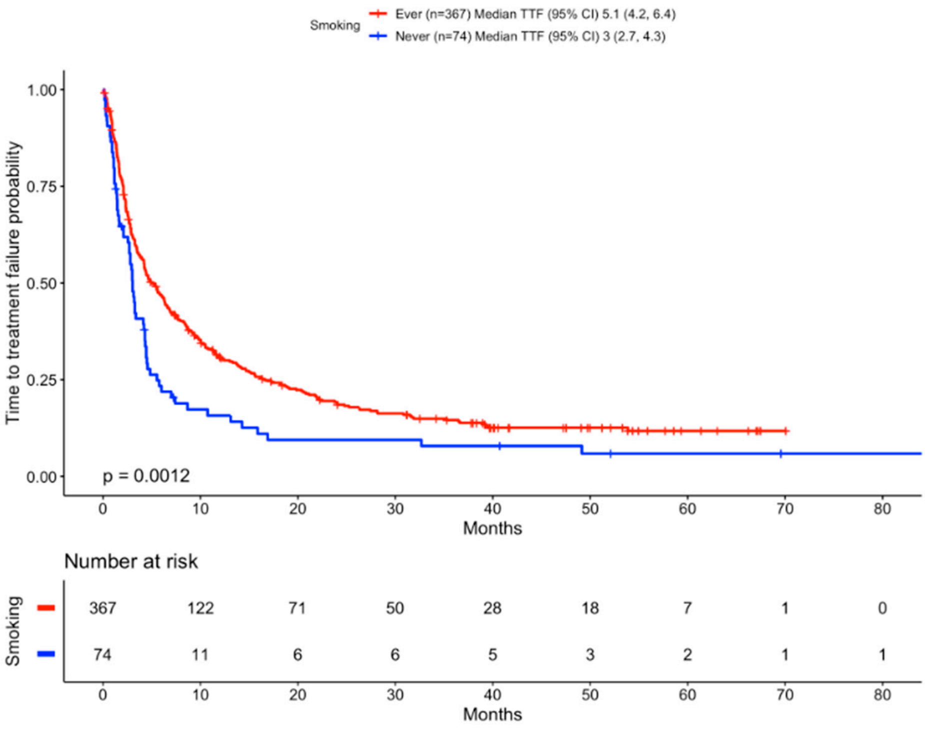

G. Combined CVI

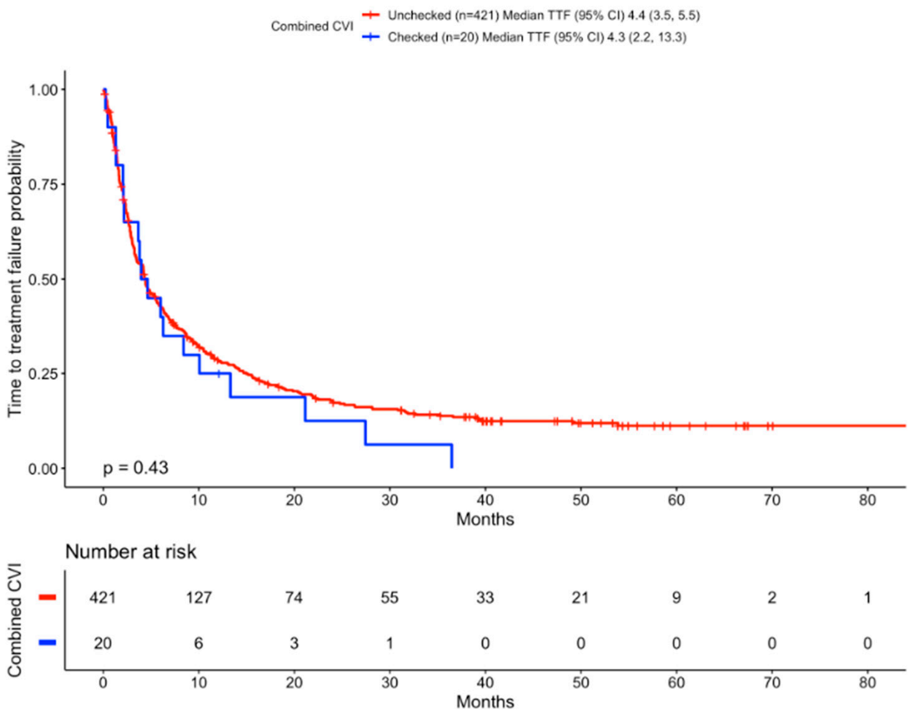

H. Number of metastatic sites

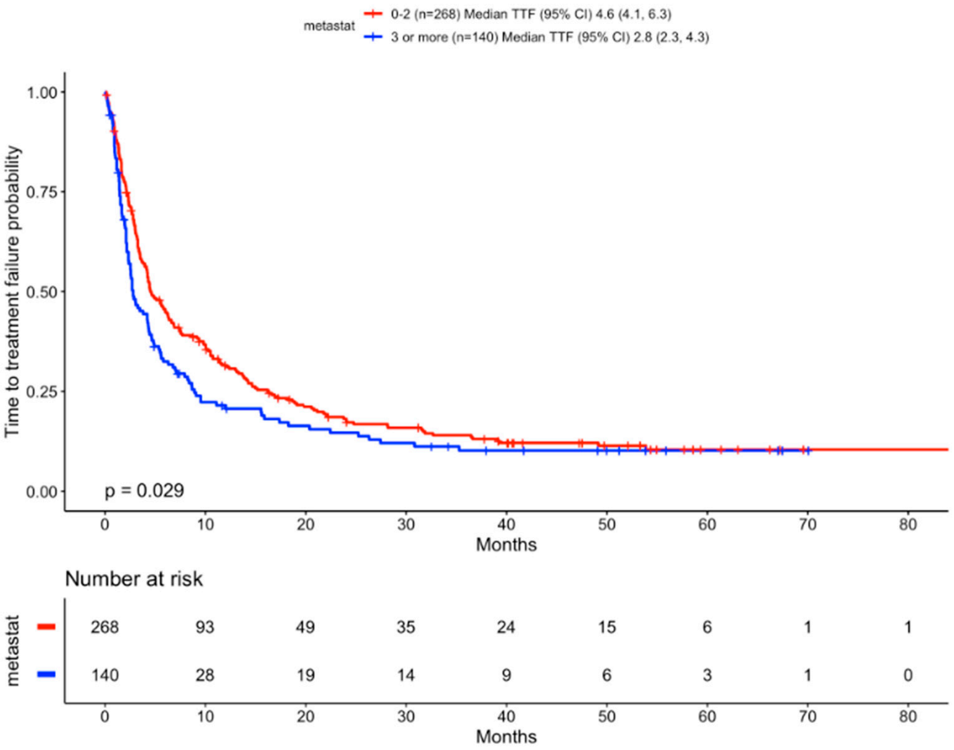

Figure S11. Time to treatment failure (TTF) probability in NSCLC patients treated with PD-L1 monotherapy by unique cohorts.

A. Age ≤75 years vs. Age >75 years

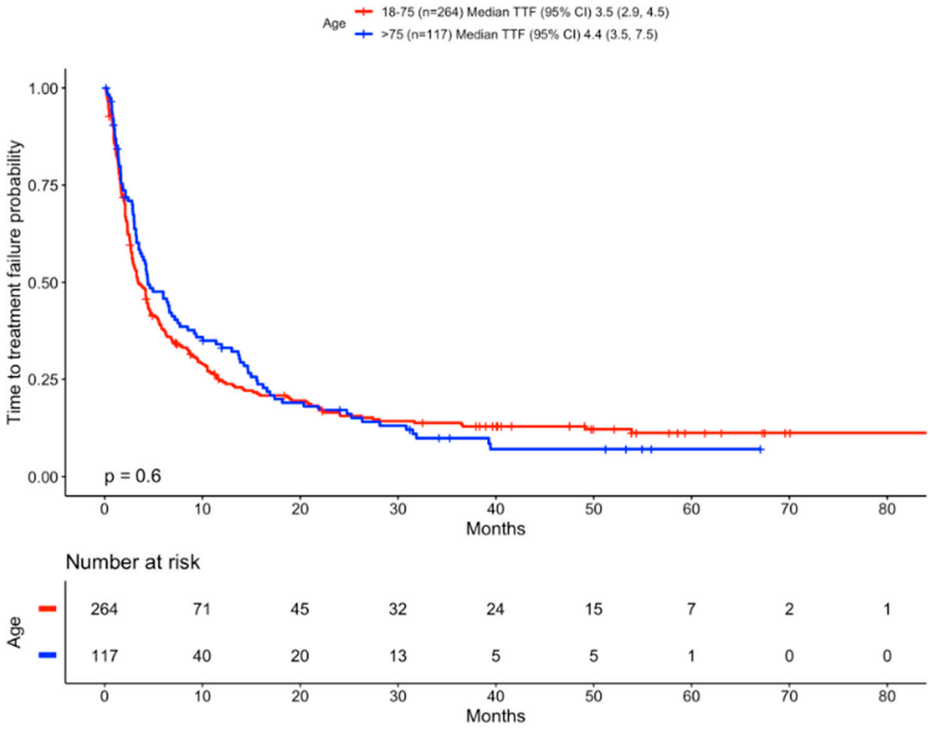

B. Race

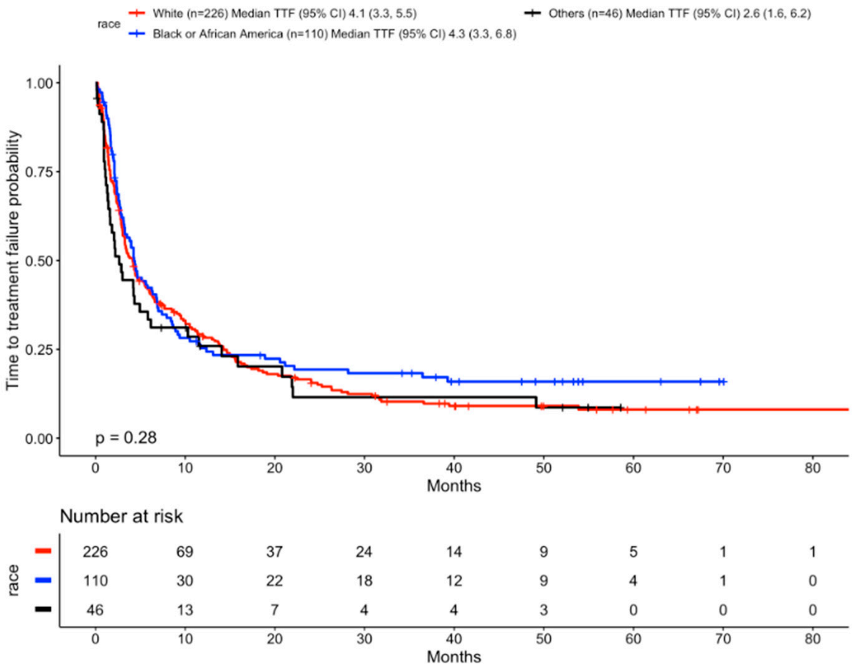

C. Gender

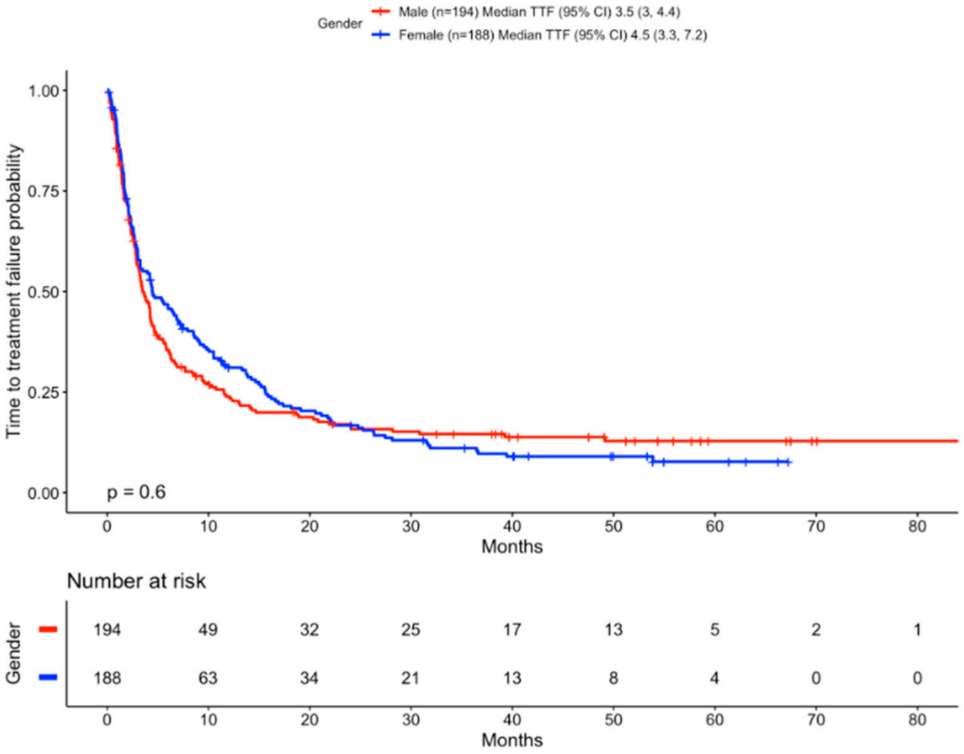

D. History of AID

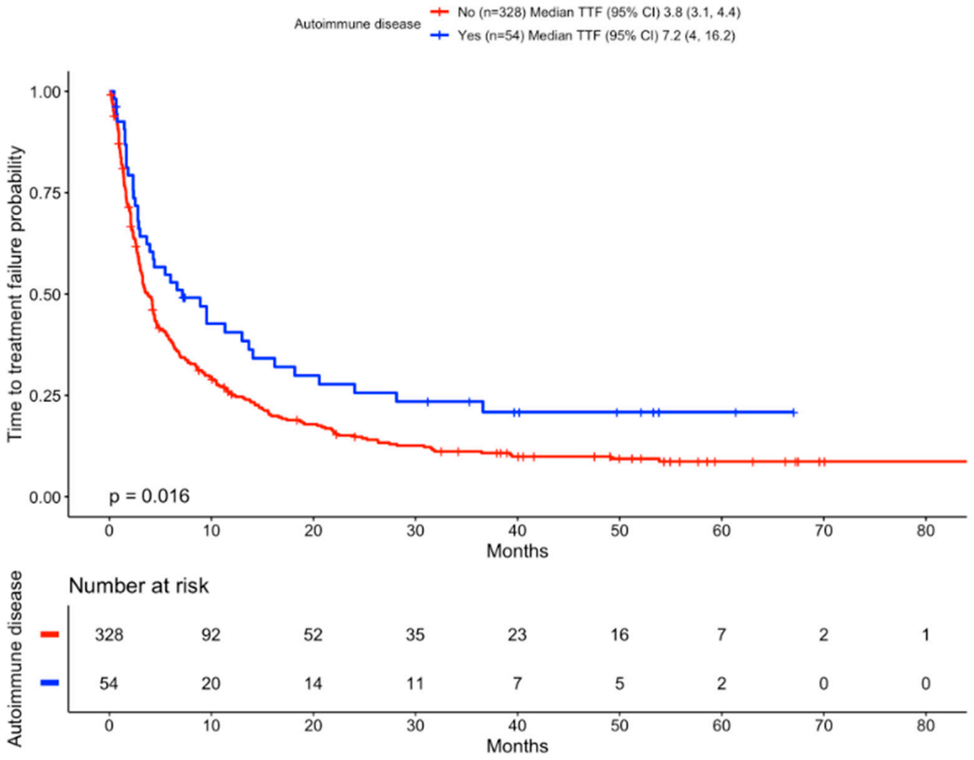

E. BMI<30 kg/m² vs. BMI ≥30 kg/m²

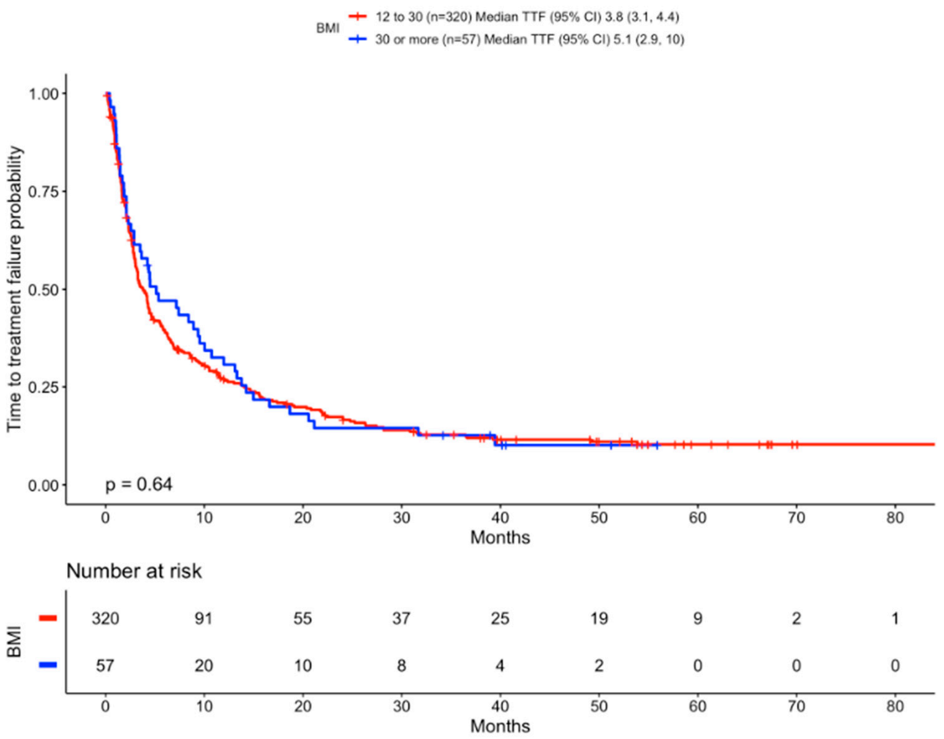

F. Smoking

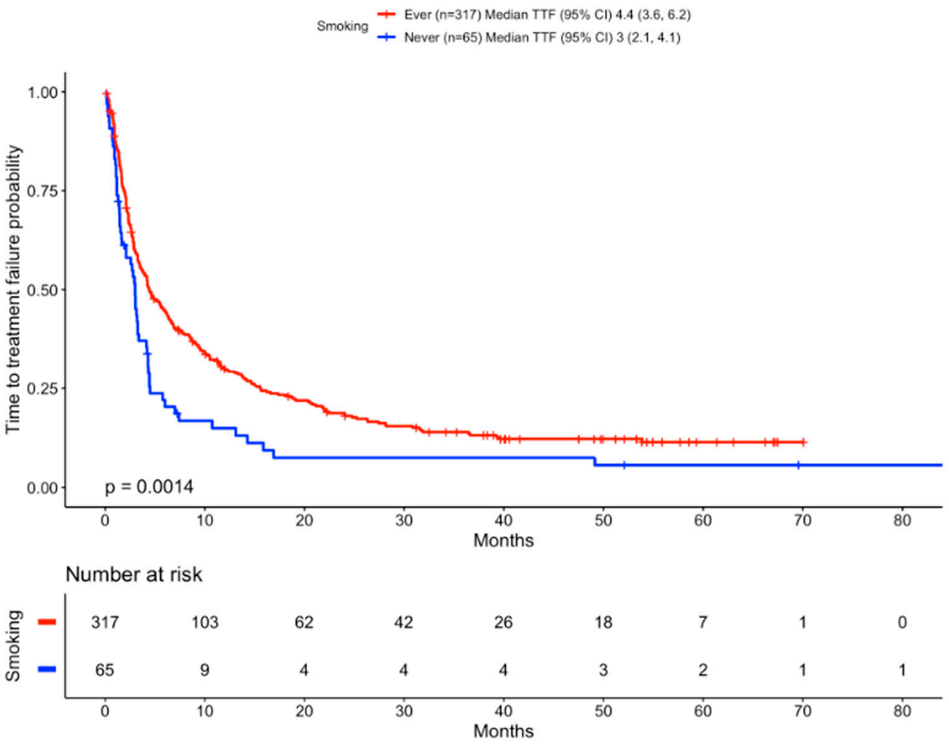

G. Combined CVI

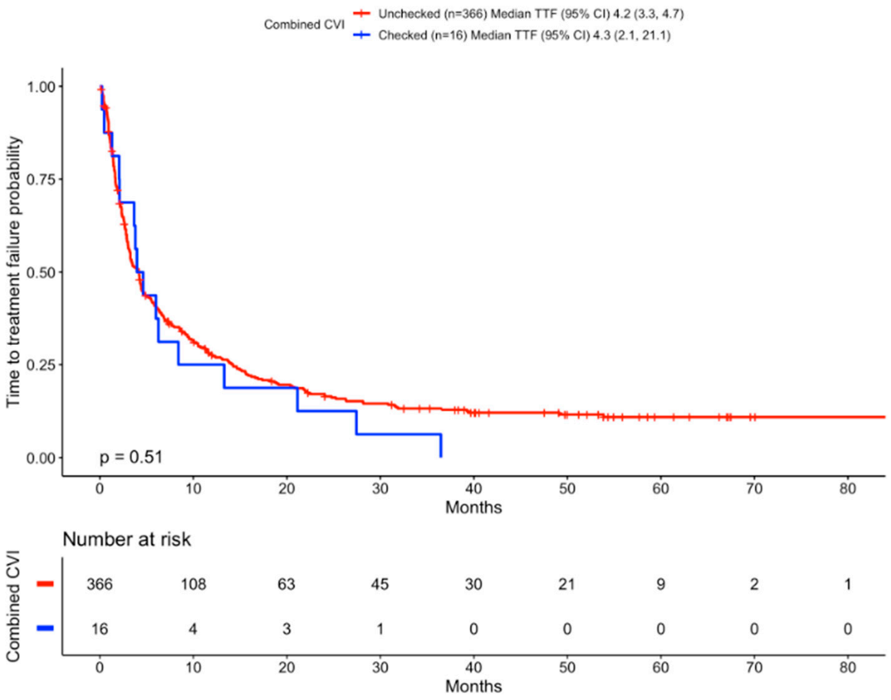

H. Number of metastatic sites

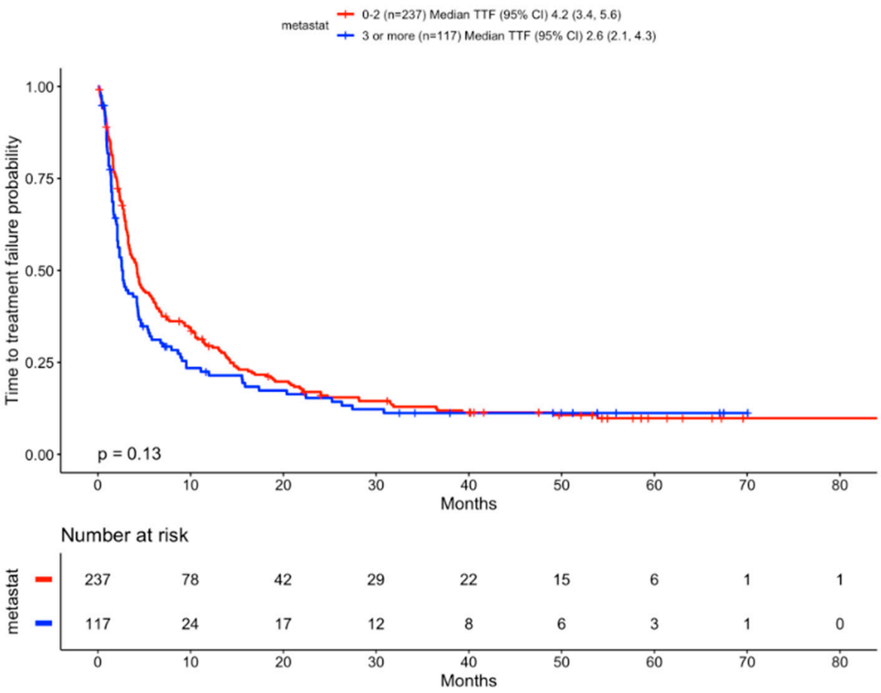

Figure S12. Time to treatment failure hazard ratio by unique patient groups of interest in White patients in the anti-PD-(L)1 monotherapy NSCLC cohort.

Variable		N	Hazard ratio	p
**Age, years**	18-75	132	Reference	
	>75	70	1.00 (0.72, 1.39)	0.992
**Gender**	Male	93	Reference	
	Female	109	0.71 (0.52, 0.96)	0.028
**Autoimmune disease**	No	169	Reference	
	Yes	33	0.67 (0.44, 1.03)	0.068
**Pre-treatment ECOG PS**	0 - 1	145	Reference	
	2 or more	57	1.60 (1.15, 2.23)	0.005
**Lines of therapy**	1	48	Reference	
	2	108	1.20 (0.83, 1.75)	0.334
	>=3	46	1.81 (1.16, 2.82)	0.009

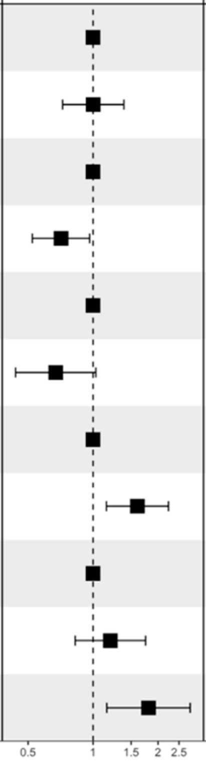

May and Hosmer goodness-of-fit test *0.* = 0.309.

Figure S13. Time to treatment failure hazard ratio by unique patient groups of interest in Black patients in the anti-PD-(L)1 monotherapy NSCLC cohort.

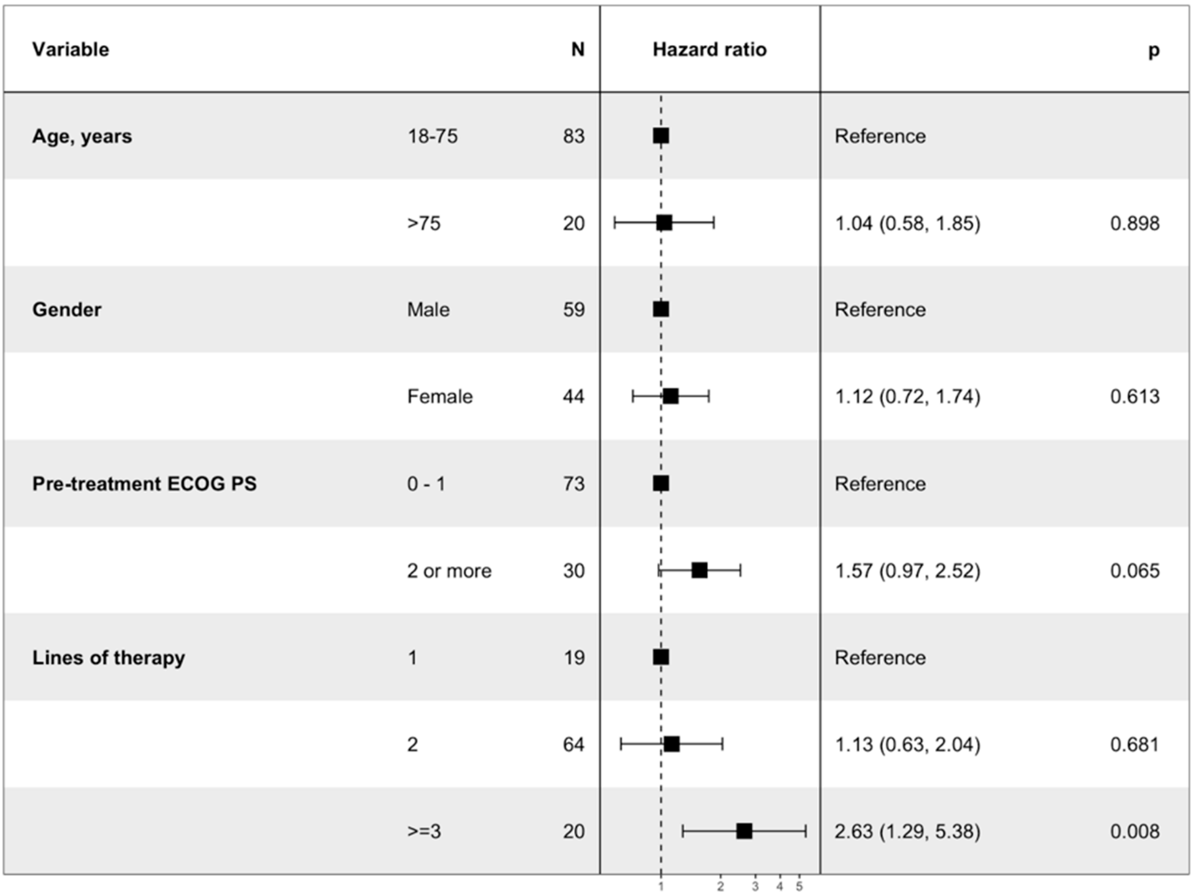

May and Hosmer goodness-of-fit test p = 0.742.

Figure S14. Overall survival hazard ratio in the entire NSCLC cohort treated with ICIs by unique cohorts.

Variable		N	Hazard ratio		p
**Age, years**	18-75	284		Reference	
	>75	112		1.08 (0.80, 1.46)	0.608
**Race**	White	234		Reference	
	Black or African America	118		0.84 (0.62, 1.13)	0.258
	Others	44		1.03 (0.66, 1.60)	0.900
**Gender**	Male	197		Reference	
	Female	199		0.88 (0.68, 1.14)	0.331
**BMI, kg/m2**	12 to 30	340		Reference	
	30 or more	56		1.05 (0.74, 1.48)	0.779
**Smoking**	Ever	325		Reference	
	Never	71		1.27 (0.91, 1.76)	0.156
**Combined Chronic Viral Infections (CVI)**	No	379		Reference	
	Yes	17		1.38 (0.77, 2.46)	0.274
**Autoimmune disease**	No	339		Reference	
	Yes	57		0.81 (0.56, 1.18)	0.275
**Pre-treatment ECOG PS**	0 - 1	297		Reference	
	2 or more	99		1.72 (1.28, 2.31)	<0.001
**Number of metastatic sites**	0-2	259		Reference	
	3 or more	137		1.36 (1.04, 1.78)	0.023
**Lines of therapy**	1	114		Reference	
	2	201		1.35 (0.99, 1.84)	0.055
	>=3	81		1.77 (1.23, 2.56)	0.002

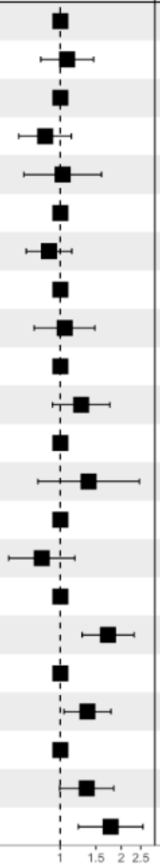

May and Hosmer goodness-of-fit test p = 0.359.
